# Role of land cover in Finland’s greenhouse gas emissions

**DOI:** 10.1007/s13280-023-01910-8

**Published:** 2023-09-07

**Authors:** Maria Holmberg, Virpi Junttila, Torsti Schulz, Juha Grönroos, Ville-Veikko Paunu, Mikko Savolahti, Francesco Minunno, Paavo Ojanen, Anu Akujärvi, Niko Karvosenoja, Pirkko Kortelainen, Annikki Mäkelä, Mikko Peltoniemi, Jouko Petäjä, Pekka Vanhala, Martin Forsius

**Affiliations:** 1 Finnish Environment Institute (SYKE), Latokartanonkaari 11, 00790 Helsinki, Finland; 2 Department of Forest Sciences, Institute for Atmospheric and Earth System Research (INAR) & Faculty of Agriculture and Forestry, University of Helsinki, Latokartanonkaari 7, P.O. Box 27, 00014 Helsinki, Finland; 3 Natural Resources Institute Finland (Luke), Latokartanonkaari 9, 00790 Helsinki, Finland

**Keywords:** Carbon sequestration, Greenhouse gas emissions, Land cover, Regional aggregation

## Abstract

We present regionally aggregated emissions of greenhouse gases (GHG) from five land cover categories in Finland: artificial surfaces, arable land, forest, waterbodies, and wetlands. Carbon (C) sequestration to managed forests and unmanaged wetlands was also assessed. Models FRES and ALas were applied for emissions (CO_2_, CH_4_, N_2_O) from artificial surfaces and agriculture, and PREBAS for forest growth and C balance. Empirical emission coefficients were used to estimate emissions from drained forested peatland (CH_4_, N_2_O), cropland (CO_2_), waterbodies (CH_4_, CO_2_), peat production sites and undrained mires (CH_4_, CO_2_, N_2_O). We calculated gross emissions of 147.2 ± 6.8 TgCO_2_eq yr^−1^ for 18 administrative units covering mainland Finland, using data representative of the period 2017–2025. Emissions from energy production, industrial processes, road traffic and other sources in artificial surfaces amounted to 45.7 ± 2.0 TgCO_2_eq yr^−1^. The loss of C in forest harvesting was the largest emission source in the LULUCF sector, in total 59.8 ± 3.3 TgCO_2_eq yr^−1^. Emissions from domestic livestock production, field cultivation and organic soils added up to 12.2 ± 3.5 TgCO_2_eq yr^−1^ from arable land. Rivers and lakes (13.4 ± 1.9 TgCO_2_eq yr^−1^) as well as undrained mires and peat production sites (14.7 ± 1.8 TgCO_2_eq yr^−1^) increased the total GHG fluxes. The C sequestration from the atmosphere was 93.2 ± 13.7 TgCO_2_eq yr^−1^. with the main sink in forest on mineral soil (79.9 ± 12.2 TgCO_2_eq yr^−1^). All sinks compensated 63% of total emissions and thus the net emissions were 53.9 ± 15.3 TgCO_2_eq yr^−1^, or a net GHG flux per capita of 9.8 MgCO_2_eq yr^−1^.

**Supplementary Information:**

The online version contains supplementary material available at 10.1007/s13280-023-01910-8.

## Introduction

Mitigation of climate change and adaptation to its consequences require both global and local actions. The international Paris Agreement (UNFCCC 2015) aims at limiting global warming to well below 2, preferably to 1.5 degrees Celsius, compared to pre-industrial levels. To achieve this long-term temperature goal, countries aim to reach global peaking of greenhouse gas (GHG) emissions as soon as possible to achieve a climate neutral world by mid-century. Consequently, the European Commission strives to achieve net-zero GHG emissions by 2050 (EC 2021), and Finland has an even more ambitious target of carbon (C) neutrality by 2035. To comply with international and regional commitments (UNFCCC 2015, EC 2018, 2021) individual countries document annually their emissions and removals in national inventory reports (e.g. Statistics Finland 2022a). The official, mandatory national inventory required by the UNFCCC, EU and the Paris agreement covers emissions and removals of GHGs from five sectors: energy, industrial processes and product use, agriculture, land use, land use change and forestry (LULUCF), and waste. The inventory reports provide essential information also for the planning and monitoring of national climate policies, including detailed information on emission trends (Statistics Finland 2023).

Land use management strategies have a large climate mitigation potential. At the same time, biodiversity conservation targets must be considered (EC 2020). According to Roe et al. (2019) measures in forestry, agriculture, wetlands, and bioenergy could contribute 15 GtCO_2_eq per year (about 30%) of the global mitigation needed by 2050 to reach the 1.5 °C target. Examples of measures in the LULUCF sector being restoring forests and drained peatlands as well as improving forest management (Roe et al. 2019). In a recent literature review on GHG emissions and removals associated with rewetting agricultural soils Bianchi et al. (2021) found a significant mitigation potential, although more information is needed on the emission factors reflecting different climate conditions and management practices. Mitigation measures are central also for national climate change roadmaps (e.g. Finnish Climate Change Panel 2019). Actors on the regional and municipal levels are often able to make decisions that affect local emissions. They are responsible for policies and management actions guiding land use. Many Finnish regions and communities have their own climate roadmaps and local level C neutrality goals (Saikku et al. 2022). Comprehensive regional-level information on the sources and sinks of GHGs from different land use forms supports regional GHG emission mitigation efforts (Buffam et al. 2011; Vanhala et al. 2016; Holmberg et al. 2021). Kangas and Ollikainen (2022) presented a policy tool promoting both climate and biodiversity targets. Kangas and Ollikainen (2023) analysed a hypothetical reform on a scheme of forest biodiversity payments for ecosystem services (PES). Supplemented with a payment for providing C benefits, this scheme would function as a new tool to achieve both the goals of the EU Biodiversity Strategy and the requirements of the LULUCF regulation. Shin et al. (2022) reviewed biodiversity conservation actions which have the largest potential for mitigation of climate change and found synergistic benefits. Smith et al. (2023) presented potential pathways towards sustainable food and land use systems in the UK, with multiple benefits for climate, biodiversity, and health.

In Finland, GHG emissions from the sectors energy, industry, agriculture, and waste management have decreased from 53.9 to 46.9 TgCO_2_eq yr^−1^ between 2015 and 2021. During the same period, for the LULUCF sector, both its emissions and the amount of carbon sequestered decreased, whereby the LULUCF net sink deteriorated from -16.7 to 0.5 TgCO_2_eq yr^−1^ (Statistics Finland 2023). Forest land covers over 75% of the land area in Finland (Niinistö et al. 2021) providing a large part of the LULUCF sink (Statistics Finland 2023). During 2015 to 2021, the net emissions, including LULUCF, have increased from 38.4 to 48.4 TgCO_2_eq yr^−1^ (Statistics Finland 2023). In 2020, Finnish net per capita emissions were 5.7 MgCO_2_eq yr^−1^ compared to the average 7.0 MgCO_2_eq yr^−1^ for 27 countries in the European Union (Eurostat 2022a).

Forestry activities, in particular harvesting, determine to a large extent the annual sink variation in the national GHG inventory. For example, a decrease of 6% in commercial fellings from 2019 to 2020 was the main cause in the increase of 27% in C sink (removal from the atmosphere) (Statistics Finland 2022a). Monni et al. (2007) reported a large uncertainty in the forest sink values. Peatlands have been drained to increase forest production and currently 4.8 Mha or more than half of Finland’s peatlands are drained (Niinistö et al. 2021), acting as sources or sinks of GHGs, depending on their fertility, vegetation cover and hydrology (Ojanen et al. 2010, 2013). Cultivated organic soils are a major source of agricultural GHG emissions although they cover only 10% of the field area in Finland (Regina et al. 2019) and offer a considerable mitigation potential (Kekkonen et al. 2019). Changes in soil organic carbon in organic forest and agricultural soils caused the largest emissions in the LULUCF sector in Finland in 2019 (10.5 Tg CO_2_eq yr^−1^) (Statistics Finland 2022a).

According to Turunen and Valpola (2020) different forms of land use of Finnish peatlands have reduced the total peat C store by 3–10% (ca. 172–510 TgC) since 1950. The largest peat C losses have occurred from forestry-drained peatlands due to their vast area, but significant losses have occurred also from agricultural peat soils, peat extraction, and other forms of peatland exploitation due to their high area-specific emissions. Finnish lakes cover ca 10% of the surface area and have been shown to be important as sources of GHGs in the landscape (Kortelainen et al. 2004, 2006, 2013, 2020). A recent study on the GHG balance of a large river basin in SW Finland reported net emission intensity 0.16 GgCO_2_eq km^−2^ yr^−1^, and per capita emissions of 5.6 MgCO_2_eq yr^−1^ (Holmberg et al. 2021).

The aims of this paper are to: (i) provide detailed, spatially explicit estimates of current GHG emission sources and sinks for main land cover categories in the landscape in mainland Finland; (ii) aggregate and calculate the net emissions for the categories; (iii) discuss the role of the different land cover categories and evaluate the uncertainties of the category-specific estimates; and (iv) discuss the variation in emissions between regions by presenting the results for 18 administrative regions covering mainland Finland. Our estimates are based on data and model parameters that represent the period 2017–2025.

## Materials and methods

### Study area

Finland is a Nordic country, stretching between roughly 60° to 70° north, and 20° to 31° east in the boreal biogeographical zone. The mean surface temperature varies from 5.9 °C in the south to − 1.3 °C in the North (Aalto et al. 2016). The south, middle and north boreal zones dominate the country mainland (Henttonen et al. 2019). Excluding the Åland Islands, country total land and inland water area amounts to 336 887 km^2^, with a population of 5.5 million (Supplementary Table S1). The Åland Islands (land area 13 300 km^2^, population 30 300) were not included in our study because we lacked data on the distribution of emissions from artificial surfaces in that region. Forestry is the dominant land cover on the mainland, with up to over 80% of land area covered by forest land in eastern regions, and high fractions of peat soils in northwestern forests (Niinistö et al. 2021). The most important agricultural areas are in the southern and western regions (Regina et al. 2019). In 2019, Finland’s GDP was EUR 251 367 million, or EUR 45 360 per capita. Forestry and agriculture each contributed EUR 4187 million and EUR 1363 million, respectively, to the total value added of the national economy (Eurostat 2022b, c). The population of mainland Finland lives mainly in densely populated areas, average degree of urbanization being 86.7% (Table S1). Uusimaa on the southern coast, with the country’s highest population density (187, Figure S1, Table S1), includes three of the country’s five most populated cities—the capital Helsinki, Espoo, and Vantaa. Other major cities are Tampere in Pirkanmaa, Oulu in North Ostrobothnia and Turku in Southwest Finland.

### GHG fluxes and emission intensities by land cover category

Average annual emissions of GHG, expressed as CO_2_eq yr^−1^, were calculated for five land cover categories: artificial surfaces (CH_4_, CO_2_, N_2_O), arable land (CH_4_, CO_2_, N_2_O), forest (CH_4_, CO_2_, N_2_O), waterbodies (CH_4_, CO_2_), and wetlands (CH_4_, CO_2_, N_2_O). Models were applied to calculate emissions from artificial surfaces, agriculture, and peat production sites (FRES, Karvosenoja 2008; ALas, Lounasheimo et al. 2020) and forest carbon balance (PREBAS, Minunno et al. 2019). The modelling approach enables scenario analysis (FRES and PREBAS), and accounting for changing climate (PREBAS). Scenario analysis results are presented in accompanying papers (Forsius et al. 2023 and Junttila et al. 2023). Area-specific emission coefficients were used to estimate emissions from cultivated cropland, waterbodies, and wetlands. When fluxes of carbon (C) or nitrogen (N) were the basis of the calculations, molar masses were utilized to convert these fluxes to CO_2_, CH_4_, or to N_2_O depending on the processes involved. The emissions were then expressed as CO_2_eq using the global warming potential (GWP_100_) values 1 (CO_2_), 28 (CH_4_) and 265 (N_2_O) (Supplementary Table S2) corresponding to the IPCC AR5 GWP values for 100 years (Myhre et al. 2013, p. 73).

Several data sources were utilised to estimate the surface areas of the land cover categories and subcategories (Table 1). The area of artificial surfaces was estimated as the sum of the CLC Level 2 classes 11 (urban fabric), 12 (industrial, commercial and transport units), and 13 (mine, dump and construction sites) (Härmä et al. 2019). For cultivated cropland area we used the reported plots for the year 2020 in the land parcel register of the Finnish Food Authority, divided into mineral and organic soils based on the digital soil map (Lilja et al. 2006). The area allocated for domestic livestock production was estimated as the difference between utilised total agricultural area and cropland area (OSF 2023a). Here, forestry land included forest land and poorly productive land, only. Unproductive forest land, forest roads and depots, and other forest land were excluded from the study. Forest area was based on the 12th Multi-Source National Forest Inventory (MS-NFI 2019) estimates (Mäkisara et al. 2022). Forest pixels (16 m) on mineral soil and drained peatland were classified using National Land Survey data (Haakana et al. 2022). Areas of rivers and lakes were obtained from the river network data set of Finnish Environment Institute. The area of peat production sites was taken as the CLC Level 4 class 4122. The area of undrained mires was based on MS-NFI 2019 and information on drainage status. The total area of this study is 9% smaller than the area of mainland Finland including inland waters (Table S1). This difference is explained by the areas that were not included here: grasslands, cultivated plots outside subsidies; unproductive forest land, forest roads, depots and other forest land.

**Table 1:** Land cover surface areas aggregated for the whole country

Land cover category	Subcategory	Area^a^ (km^2^)	Fraction (%) of landcover class	Fraction (%) of total area
Artificial surfaces^a,b^ total		7973		3
Arable land	Domestic livestock production^c^	1986	9	
Arable land	Organic soil annual crops^d^	958	4	
Arable land	Organic soil perennial crops^d^	1212	5	
Arable land	Mineral soil all crops^d^	18 265	81	
Arable land	Field cultivation total^e^	20 435	91	
Arable land^a,c,d,e^ total		22 537	100	7
Forest	Forest mineral soil^f^	174 405	83	
Forest	Forest drained peatland^f^	36 725	17	
Forest^a,^^f^ total		211 130	100	69
Waterbodies	Rivers^g^	1388	4	
Waterbodies	Lakes^h^	32 510	96	
Waterbodies^a,^^g,h^ total		33 896	100	11
Wetland	Peat production sites^i^	1026	3	
Wetland	Undrained mires^j^	30 508	97	
Wetland^a,i,j^ total		31 534	100	10
Total^a^ land cover area		306 954		100

^a^Areas refer to mainland Finland (excluding Åland). Grasslands and barren hill tops are not included

^b^CLC Level 2 classes 11, 12 and 13

^c^Estimated from utilised total agricultural area and cropland area

^d^Cropland area

^e^Estimated same as Cropland area

^f^Forest land and poorly productive forest land on mineral soil and drained peatland MS-NFI 2019

^g^For rivers less wide than 5 m, average width 3.5 m assumed

^h^Lakes larger than 1 ha included

^i^CLC Level 4 class 4122

^j^MS-NFI 2019, Luke peatland mask

The spatial resolution for each land cover category was determined by the main data sources used (Supplementary Table S4). For emissions from artificial surfaces, emissions from individual plants were combined with emissions from the so-called area sources to 250 m resolution. Emissions from cropland were calculated in 250 m resolution using information on mineral/organic soil classification of the national digital soil map (Lilja et al. 2006, 2017). Results from forest calculations were available in 16 m resolution and transformed to 250 m resolution. Emissions from waterbodies were calculated for individual lakes and river stretches and transformed to 250 m resolution. Emissions from undrained mires were calculated in 250 m resolution using information on drainage status (Luke), forest soil and type classification available in 16 m resolution (MS-NFI 2019, Mäkisara et al. 2022). In addition to causing GHG emissions, forests and undrained mires also sequestered C from the atmosphere. Net emissions were calculated by subtracting the sequestration flux from the emission flux. The sequestration flux is given as negative numbers in tables that show the net emissions (Tables 2 and 3).

**Table 2:** GHG fluxes (TgCO_2_eq yr^−1^) and flux intensity (GgCO_2_eq km^−2^ yr^−1^) from different emission sources

Land cover	Source or sink	CH_4_ (TgCO_2_eq yr^−1^)	CO_2_ (TgCO_2_eq yr^−1^)	N_2_O (TgCO_2_eq yr^−1^)	Total (± uncertainty) (TgCO_2_eq yr^−1^)	Intensity^b,c^ (GgCO_2_eq km^−2^ yr^−1^)
Artificial surfaces	Industrial processes total	0.00	8.51	0.00	8.51 (± 0.30)	1.07
Artificial surfaces	Energy production—waste and other solids	0.00	6.49	0.00	6.49 (± 0.13)	0.81
Artificial surfaces	Energy production—peat	0.00	5.76	0.04	5.80 (± 0.20)	0.73
Artificial surfaces	Energy production—gaseous fuels	0.00	5.72	0.00	5.72 (± 1.59)	0.72
Artificial surfaces	Energy production—liquid fuels	0.00	1.67	0.00	1.67 (± 0.07)	0.21
Artificial surfaces	Energy production—biomass	0.01	0.00	0.09	0.10 (± 0.05)	0.01
Artificial surfaces	Energy production total	0.01	19.63	0.13	19.77 (± 0.59)	2.48
Artificial surfaces	Road traffic	0.00	10.37	0.00	10.37 (± 0.31)	1.30
Artificial surfaces	Machinery and off-road transport	0.00	3.16	0.00	3.16 (± 0.19)	0.40
Artificial surfaces	Waste management	1.88	0.00	0.10	1.98 (± 0.70)	0.25
Artificial surfaces	Residential and other small-scale combustion	0.19	1.66	0.03	1.88 (± 0.44)	0.24
Artificial surfaces total emission		2.08	43.34	0.26	45.68 (± 1.97)	5.73
Arable land	Domestic livestock production	2.80	0.00	0.24	3.04 (± 2.28)	1.53
Arable land	Field cultivation	0.00	0.20	3.22	3.43 (± 2.57)	0.17
Arable land	Organic soil annual crops	0.00	2.78	0.00	2.78 (± 0.48)	2.90
Arable land	Organic soil perennial crops	0.00	2.53	0.00	2.53 (± 0.67)	2.09
Arable land	Mineral soil all crops	0.00	0.47	0.00	0.47 (± 0.25)	0.03
Arable land total emission		2.80	5.98	3.47	12.24 (± 3.54)	0.55
Forest	Timber harvest, forest on mineral soil	0.00	44.75	0.00	44.75 (± 3.12)	0.26
Forest	Timber harvest, forest on drained peatland	0.00	8.84	0.00	8.84 (± 0.49)	0.24
Forest	Timber harvest total	0.00	53.60	0.00	53.60 (± 3.16)	0.25
Forest	Energywood harvest, forest on mineral soil	0.00	5.23	0.00	5.23 (± 0.89)	0.03
Forest	Energywood harvest, forest on drained peatland	0.00	0.99	0.00	0.99 (± 0.14)	0.03
Forest	Energywood harvest total	0.00	6.22	0.00	6.22 (± 0.90)	0.03
Forest	Harvest total	0.00	59.82	0.00	59.82 (± 3.29)	0.28
Forest	Drained peatland, soil emissions	0.35	0.00	1.44	1.79 (± 3.52)	0.05
Forest total emission		0.35	59.82	1.44	61.61 (± 4.81)	0.29
Forest sink	Forest ecosystems on mineral soil^a^		− 79.87		− 79.87 (± 12.20)	− 0.38
Forest sink	Forest ecosystems on drained peatland^a^		− 9.41		− 9.41 (± 6.10)	− 0.04
Forest total sink			− 89.28		− 89.28 (± 13.70)	− 0.42
Forest net emission		0.35	− 29.47	1.44	− 27.68 (± 10.30)	− 0.13
Waterbody	Rivers	0.00	7.33	0.00	7.33 (± 1.20)	5.28
Waterbody	Lakes	0.86	5.16	0.00	6.02 (± 1.50)	0.19
Waterbody total emission		0.86	12.49	0.00	13.35 (± 1.92)	0.39
Wetland	Peat production	0.06	1.80	0.09	1.95 (± 0.39)	1.90
Wetland	Undrained mires, soil emissions	11.48		0.84	12.32 (± 1.79)	0.40
Wetland total emission		11.54	1.80	0.93	14.27 (± 1.84)	0.45
Wetland sink	Undrained mires ecosystems^a^		− 3.91		− 3.9 (± 0.40)	0.00
Wetland net emission		11.54	− 2.11	0.93	10.36 (± 4.40)	0.34
Total emission to the atmosphere		17.63	123.43	6.10	147.16 (± 6.83)	0.48
Net emission to the atmosphere		17.63	30.23	6.10	53.9 (± 15.30)	0.18

^a^Excluding sequestration of CH_4_

^b^Intensity as emissions (or sinks) divided by land cover area

^c^For Artificial Surfaces, all sources emissions allocated to the whole area

**Table 3:** Emission sources and sinks aggregated for each region

Region Code	Region	Region area (land and inland water)^a^ (km^2^)	Region total emission (TgCO_2_eq yr^−1^)	Region total sink (TgCO_2_eq yr^−1^)	Region net emission (TgCO_2_eq yr^−1^)	Region average net emission intensity (GgCO_2_eq km^−2^ yr^−1^)	Region net emission per capita (MgCO_2_eq yr^−1^)
1	Uusimaa	9569	16.973	− 3.614	13.360	1.40	7.8
2	Southwest Finland	10 914	6.989	− 4.069	2.920	0.27	6.0
4	Satakunta	8269	5.787	− 2.897	2.890	0.35	13.5
5	Kanta-Häme	5708	3.973	− 1.740	2.232	0.39	13.1
6	Pirkanmaa	15 550	8.827	− 5.386	3.441	0.22	6.5
7	Päijät-Häme	6942	4.005	− 2.231	1.774	0.26	8.6
8	Kymenlaakso	4948	3.633	− 1.393	2.240	0.45	13.9
9	South Karelia	6872	4.531	− 1.694	2.837	0.41	22.5
10	South Savo	17 099	8.072	− 4.102	3.969	0.23	30.1
11	North Savo	21 078	9.542	− 6.283	3.259	0.15	13.1
12	North Karelia	22 903	7.902	− 6.728	1.174	0.05	7.2
13	Central Finland	19 012	8.732	− 6.564	2.168	0.11	8.0
14	South Ostrobothnia	14 356	7.247	− 4.878	2.368	0.16	12.3
15	Ostrobothnia	7580	4.824	− 2.939	1.885	0.25	10.7
16	Central Ostrobothnia	5224	2.550	− 1.585	0.965	0.18	14.2
17	North Ostrobothnia	39 194	18.660	− 10.333	8.328	0.21	20.0
18	Kainuu	22 688	5.537	− 7.292	− 1.754	− 0.08	− 24.6
19	Lapland	98 982	19.372	− 19.498	− 0.126	− 0.001	− 0.7
	Total	336 887	147.157	− 93.226	53.931	0.16	9.8

^a^National Land Survey of Finland. 2022

For each land cover category, the emissions were aggregated to the country level. The spatial locations of the origin of the emissions were used to aggregate the emissions to each of the administrative regions of mainland Finland (Fig. S1). Emission intensities (GgCO_2_eq km^−2^ yr^−1^) were calculated by dividing the net emissions (TgCO_2_eq yr^−1^) from each land cover category by the area of each category (km^2^). Average net GHG intensity was calculated as total net emissions divided by total area. On the regional level, net GHG by land cover category and region average net emission intensity were obtained using the corresponding regional net emissions, regional land cover areas, and total regional areas. Net GHG per capita was calculated using the total population in the 18 regions 31.12.2020 (5 503 664, Statistics Finland 2022b).

### Artificial surfaces

Emissions from artificial surfaces are caused by fuel combustion, industrial processes, and waste management. Emissions from fuel combustion and industrial processes were calculated with the FRES (Finnish Regional Emission Scenario) model (Karvosenoja 2008) to be consistent with another study (Forsius et al. 2023) in this issue. Industrial processes are large facilities where emissions are formed due to other activities than combustion of fuels for energy. For them, CO_2_ emissions reported by the operators to the national YLVA database (Compliance Monitoring Data system) are used as such for 2019.

FRES is a scenario model, where emission calculations are based on fuel use (or other) activities, emission factors and possible emission reduction technologies. It has a database of major industrial-sized facilities, called point sources, for which CH_4_, CO_2_ and N_2_O emissions are calculated individually. Calculation is based on representative fuel mixes, annual operating hours, and combustion technologies for each plant. Smaller and more numerous fuel combustion activities like traffic and households are called area sources. The model includes data on the technology, age, and fuel of, e.g. the vehicle fleet and residential heating appliances. Present-day emissions were based on the latest national fuel use data (Statistics Finland 2021) and represent the year 2019. For biogenic fuels, CO_2_ emissions were not included to avoid double-counting.

Emissions from waste management were calculated with the ALas model (Lounasheimo et al. 2020, see Supplementary Information). The ALas model is a tool for regional GHG calculation and was here used for 309 Finnish municipalities for the year 2019. Since ALas follows the same calculation principles as the national inventory report, total Finnish emissions from the waste sector match those reported in Statistics Finland (2022a).

Emissions originating from so-called area sources in artificial surfaces were aggregated to six sectors: road traffic (CO_2_), machinery and off-road transport (CO_2_), residential and other small-scale combustion (CH_4_, CO_2_, N_2_O), and waste management (CH_4_, N_2_O). Emissions from the waste sector were estimated for solid waste disposal (CH_4_), biological treatment of solid waste (CH_4_, N_2_O) and wastewater treatment (CH_4_, N_2_O).

Spatial distribution of all emissions from artificial surfaces was done with the FRES model. For area sources the FRES model uses proxies on 250 m × 250 m resolution for the distributions (Supplementary Table S3) (Paunu et al. 2013; Karvosenoja 2008). For example, road traffic emissions are distributed on the road network based on modelled and measured traffic volumes and shares of heavy- and light-duty vehicles, and agricultural emissions to fields based on field area and animal counts. FRES outputs of point source emissions were available as municipal sums that were aggregated to the level of 18 administrative regions. Since the FRES model was used for spatial allocation of all emissions from artificial surfaces, those estimates are subsequently referred to as FRES model outputs.

FRES gridded outputs of area source emissions were available on 250 m resolution covering mainland Finland. As the surface area of these grid cells was larger than the actual area of the artificial surfaces from which the emissions originated, the Corine Land Cover update for 2018 was used for the artificial surface area (Härmä et al. 2019). The estimation of uncertainty of emissions from artificial surfaces was based on source and GHG -level uncertainty intervals. The uncertainty intervals were results of activity data and emissions coefficient uncertainties (Supplementary Table S4) (Statistics Finland 2022a). We report quantitative uncertainty estimates only on country level.

### Arable land

Emissions from arable land are caused by agricultural activities such as domestic livestock production and field cultivation. Agricultural emissions were estimated for enteric fermentation (CH_4_), manure management (CH_4_, N_2_O), agricultural soils (N_2_O), field burning of agricultural residues (CH_4_, N_2_O), liming (CO_2_) and urea application (CO_2_), corresponding to the sectors 3.A, 3.B, 3.F, 3.G, 3.H, 3D.a, and 3.D.b in the Common Reporting Format of the UNFCCC. Emissions from agriculture were calculated with the ALas (Regional Calculation) model (Lounasheimo et al. 2020, Supplementary Information on ALas, Supplementary Table S5). Information on the number of livestock in each municipality is input to the calculations. Emissions from field cultivation use information on the cultivated area of different crops in each municipality and the crop yield in each region, as well as national level usage of agricultural liming material, urea, mineral nitrogen fertilizer and municipal sewage sludge. The FRES model was used to distribute the arable land emissions to 250 m × 250 m pixels throughout the country.

Soil CO_2_ emissions from cropland were estimated using area-based emission coefficients representing emissions caused by cultivation of crops on mineral and organic soils (Supplementary Table S5) (Statistics Finland 2022a). The emissions were calculated for each cropland field parcel using separate emission coefficients for annual and perennial crops on mineral soil (CO_2_), annual crops on organic soil (CO_2_), and perennial crops on organic soil (CO_2_). Field plots were classified into those on organic and mineral soils using the digital soil map of Finland (Lilja et al. 2006, 2017). We defined organic soils as soil bodies classified as Gleyic Podzols, Umbric Gleysols, and Fibric/Terric Histosols in the digital soil map (Lilja et al. 2006). For the analyses we included as cultivated plots cropland used for arable crops, cultivated hay and pasture, annual and permanent horticultural crops, greenhouses, and kitchen gardens as reported for the year 2020 in the land parcel register of the Finnish Food Authority. Cultivated plots were divided into cropland growing annual or perennial crops, with arable crops, and annual horticultural or kitchen garden crops constituting the annuals. For crops cultivated on mineral soils, the emission coefficients for carbon emissions were calculated as averages of the 2010–2020 areal emission coefficients for southern and northern Finland given in the national inventory report (Statistics Finland 2022a). Most of the regions (1 to 16) are considered southern (175 591 km^2^), while three regions 17, 18, and 19, are northern (160 848 km^2^). Uncertainties of cropland emissions were estimated based on the standard deviations given for the emission coefficients (Table S5).

### Forest

Carbon sequestration in forests was due to the estimated gross primary production of trees and ground vegetation, including herbaceous plants. Emissions of GHG from forests were estimated by accounting for harvested biomass (CO_2_), decomposition of harvest residues, litter, and soil organic matter, and area-specific emission coefficients for drained peatland (CH_4_, CO_2_, N_2_O). Carbon sequestration in forest biomass was simulated with a process-based forest growth model PREBAS (Minunno et al. 2016, 2019), using the harvest scenario BaseHarv and forest data as specified in Junttila et al. (2023) and Mäkelä et al. (2023). PREBAS is initialized using forest structural variables (i.e. average height of the stand, average diameter at breast height, basal area). The model is initialized for the three main species in Finland: Scots pine, Norway spruce and silver birch. Information on the initial state of the forest is based on data from the multi-source national forest inventories (MS-NFI), that provide forest variables at 16 m resolution. Regional harvesting intensities are modelled as annual levels of roundwood (OSF 2023b) and energywood (additional energywood based on harvest residues after roundwood, OSF 2023c) (Junttila et al. 2023; Mäkelä et al. 2023).

Harvested biomass was calculated separately for timber and energywood. Wood products were not included in the calculation, meaning that all harvested C was immediately considered as emissions. The model calculations by PREBAS were available separately for forest on mineral soil, and on drained peatland. In forested mineral soils, decomposition of harvest residues, soil organic matter and litter in forests were estimated with the soil carbon model YASSO07 (Liski et al. 2005), which together with NPP calculated by PREBAS give the net ecosystem exchange of forests on mineral soils (NEE). In drained forested peatland the soil emissions of CH_4_, CO_2_ and N_2_O (Junttila et al. 2023) were estimated with mean empirical emission coefficients (Table S11b, Ojanen et al. 2010; Ojanen and Minkkinen 2019; Minkkinen et al. 2020). Forests on undrained peat soils were excluded from the PREBAS simulation, as we do not have a model that would account for tree growth in undrained peatlands. Such simulations would require a model of water table depth combined with a model of tree growth response to water table. Undrained forest C balance was included in the overall calculation but using simple empirical estimates (see Wetlands section). The PREBAS forest results were available as annual averages for the period 2017–2025 (Junttila et al. 2023). Uncertainties of the calculation approach were estimated through Monte Carlo simulations (Junttila et al. 2023) that accounted for different sources of uncertainty: model inputs, management scenarios, climatic scenarios, and model parametric uncertainty.

### Water bodies

Emissions of GHG from lakes (CO_2_, CH_4_) and rivers (CO_2_) were calculated using area-specific empirical emission coefficients (Holmberg et al. 2021). Lake emissions were calculated for five different size classes of lakes: 0.01–0.1 km^2^, 0.1–1 km^2^, 1–10 km^2^, 10–100 km^2^ and larger than 100 km^2^. Lake emissions included CO_2_ evasion (Kortelainen et al. 2006), CH_4_ diffusion (Juutinen et al. 2009) and CH_4_ ebullition (Bastviken et al. 2004) (Supplementary Table S6). The impact of emergent macrophytes *Phragmites australis* and *Equisetum fluviatile* on CH_4_ fluxes from lakes was also considered (Bergström et al. 2007; Bergström 2011; Juutinen et al. 2003) (Supplementary Table S7). River CO_2_ emissions were estimated with area-specific emission coefficients (Supplementary Table S8) (Humborg et al. 2010). The empirical emission coefficients for GHG fluxes from waterbodies were considered to represent an estimate for current conditions, although they were based on data obtained from studies conducted at several times (Juutinen et al. 2003; Bastviken et al. 2004; Kortelainen et al. 2006; Bergström 2011; Bergström et al. 2007; Juutinen et al. 2009; Humborg et al. 2010). Uncertainties for waterbody emissions were estimated using standard deviations given for the emission coefficients (Supplementary Table S6, Vanhala et al. 2016). Spatial information on waterbodies was obtained from the river network data set by Finnish Environment Institute, which provides lakes as polygons, rivers wider than 5 m as polygons, and rivers < 5 m wide as lines. For rivers < 5 m wide, an average width of 3.5 m was assumed.

### Wetlands

Emissions from wetlands are caused by peat production and decomposition of undrained peat soils. Emissions from peat production (CH_4_, CO_2_, N_2_O) were calculated at the national level with the emission factors used in the national GHG inventory (Supplementary Table S9) (Statistics Finland 2019) and allocated to 250 m × 250 m pixels by FRES as explained for the gridded artificial surfaces emissions. The GHG balance of wetlands was estimated for the ecosystems of undrained peat soils. The calculations were based on reported empirical emission coefficients representing the net balance of both soil and vegetation on undrained mires of different characteristics (Sallantaus 1994; Turunen et al. 2002; Minkkinen and Ojanen 2013; Minkkinen et al. 2020). Undrained mires were grouped into four classes: Productive forested mires; Sedge fens; Other open and sparsely treed fens; Ombrotrophic bogs (Supplementary Table S10). The grouping was based on the forest productivity classification of the site (productive, poorly productive, unproductive), site fertility class and site main class (spruce mire, pine mire, or open bog) based on data from the 2019 multi-source national forest inventory (Mäkisara et al. 2022). The emission coefficients for CH_4_ and CO_2_ were calculated following the method of Turunen et al. (2002) using data of Minkkinen and Ojanen (2013) to estimate the average long-term apparent rate of carbon accumulation in undrained mires with varying vegetation, soil and hydrological characteristics. Furthermore, a constant net leaching of C was assumed (Sallantaus 1994; Minkkinen and Ojanen 2013). For nitrous oxide emission, coefficients were calculated based on the results of Minkkinen et al. (2020) (Supplementary Table S11a). The empirical emission coefficients for undrained mires were derived from data collected at various times (Sallantaus 1994; Turunen et al. 2002; Minkkinen and Ojanen 2013; Minkkinen et al. 2020), and represent the best available estimates for current conditions. Uncertainties for wetlands emissions were estimated using standard deviations given for the emission coefficients (Supplementary Tables S11a, S11b).

## Results

**Fig. 1:**
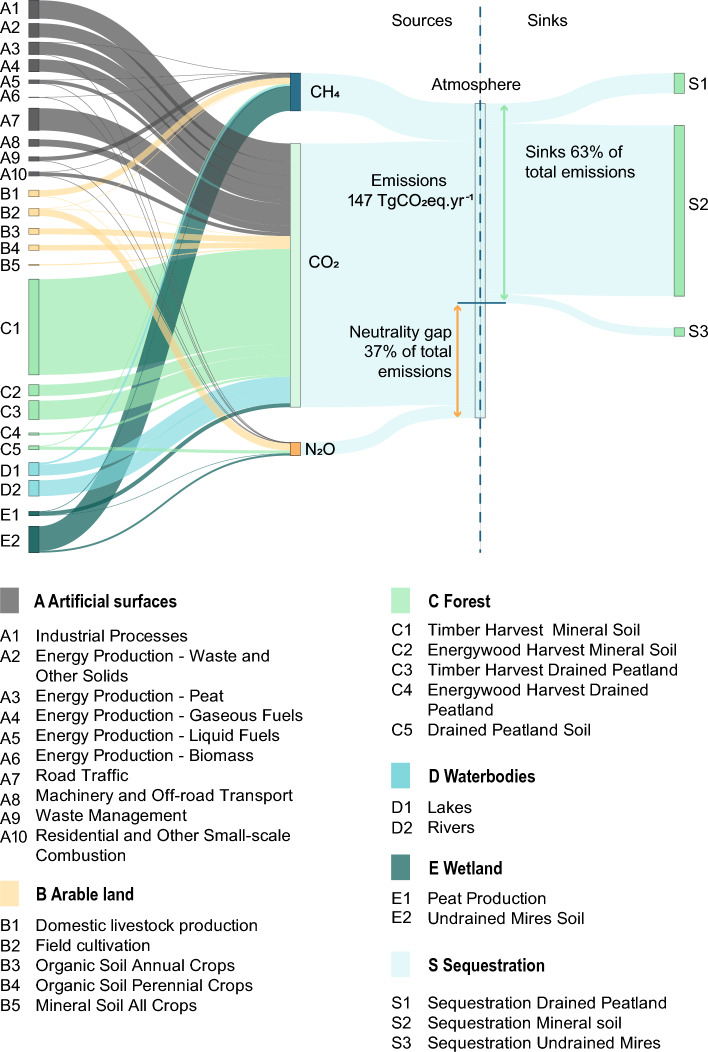
Summary of GHG fluxes from main land cover categories in 18 regions of mainland Finland (TgCO_2_eq yr^−1^). Emissions to the atmosphere (TgCO_2_eq yr^−1^) of CH_4_, CO_2_, and N_2_O, and sequestration of C(TgCO_2_eq yr^−1^) from the atmosphere to ecosystems (Table 2). The flow direction is from left to right, and the width of each line represents the flow rate of each source (to the left of Atmosphere) or sink (to the right). This graph does not reflect the national GHG inventory, as the calculation methods differ (especially forests). Furthermore lakes, rivers and undrained mires are not included in the national inventory, but here shown to display the fluxes of all main land cover categories

Total emissions to the atmosphere were 147.2 ± 6.8 TgCO_2_eq yr^−1^ and after subtracting a total sequestration of 93.2 ± 13.7 TgCO_2_eq yr^−1^ the net remaining emissions were 53.9 ± 15.3 TgCO_2_eq yr^−1^ (Table 2). This means that the remaining gap to reach climate neutrality in Finland currently amounts to 37% of emissions (Fig. 1) The uncertainty of the sink estimate was much higher than that of the emissions. Our mean estimate of net emissions was higher than the 2021 value in the national report 48.4 TgCO_2_eq yr^−1^ (Statistics Finland 2023). Accounting for the uncertainty, however, brings our low estimate (38.6 TgCO_2_eq yr^−1^) closer to the national value. The difference with respect to the nationally reported GHG consists of our estimates of the waterbody and soil emissions from unmanaged wetlands 13.4 ± 2.7 TgCO_2_eq yr^−1^ and 12.3 ± 1.8 TgCO_2_eq yr^−1^. Because we included these land cover classes in our calculations, the average per capita net emission for the 18 regions (9.8 MgCO_2_eq yr^−1^) was 1.7 higher than the official per capita net GHG for Finland in 2020 (Eurostat 2022a). Averaged for the 18 regions over the area of all the land cover classes, the net emission intensity was 0.18 GgCO_2_eq km^−2^ yr^−1^, which is 15% higher than the net emission intensity 0.16 GgCO_2_eq km^−2^ yr^−1^ calculated from 2019 reported values (Statistics Finland 2022a).

Emissions from artificial surfaces amounted to 45.7 ± 5.7 TgCO_2_eq yr^−1^, or 31% of the country total GHG emissions (Table 2). The area of artificial surfaces, however, covered only 3% of the total area in the 18 regions (Table 1). The largest emissions from artificial surfaces were caused by energy production (19.8 ± 0.6 TgCO_2_eq yr^−1^), road traffic (10.4 ± 0.3 TgCO_2_eq yr^−1^) and industrial processes (8.5 ± 0.3 TgCO_2_eq yr^−1^). The emissions from energy production with gaseous fuels were the most uncertain (5.72 ± 1.59, Table 2). Emission intensity for all artificial surfaces was 5.73 GgCO_2_eq km^−2^ yr^−1^ (Table 2).

Emissions from arable land were 12.24 ± 3.54 TgCO_2_eq yr^−1^, or 8% of the country total GHG emissions, with an overall emission intensity of 0.55 GgCO_2_eq km^−2^ yr^−1^ (Table 2). Roughly half of the arable land emissions (53%) came from domestic livestock production and field cultivation. The rest of the arable land emissions were CO_2_ from cropland on organic (43%) and mineral soils (4%). The organic cropland area was 9% of total arable land. Total utilized agricultural land area was 7% of the total area for the 18 regions.

A visual summary of the GHG fluxes from the different land cover classes to the atmosphere, and sequestration of C from the atmosphere (NEE), clearly shows the major role of forest both as a source and a sink (Fig. 1; Table 2). The losses of C in forest harvesting constituted the largest source in the LULUCF sector, in total 59.8 ± 3.3 TgCO_2_eq yr^−1^. Harvest emissions were divided into 44.7 ± 3.1 TgCO_2_eq yr^−1^ and 8.8 ± 0.5 TgCO_2_eq yr^−1^ from timber on mineral and drained peat soil, respectively; as well as 5.2 ± 0.9 TgCO_2_eq yr^−1^ and 1.0 ± 0.1 TgCO_2_eq yr^−1^ from energy wood on mineral and drained peat soil, respectively. Emissions of CH_4_ (0.4 ± 0.2 TgCO_2_eq yr^−1^) and N_2_O (1.4 ± 0.3 TgCO_2_eq yr^−1^) from drained peat soil increased the forest emissions to 61.6 ± 4.8 TgCO_2_eq yr^−1^ altogether, or 42% of the country total. Relative forest area was 69% (Table 1). The C sequestration in forest ecosystems (NEE) also dominated the balance (89.3 ± 13.7 TgCO_2_eq yr^−1^), or 93% of total sequestration (Table 2). Forest C sequestration gave a net forest sink of -27.7 TgCO_2_eq yr^−1^, or -0.13 GgCO_2_eq km^−2^ yr^−1^, which is about 30% larger than -0.10 GgCO_2_eq km^−2^ yr^−1^ which can be calculated from the 2019 values in the national inventory (Statistics Finland 2022a). The uncertainty in the total sink estimate was large compared to the total emission uncertainty.

Emissions from waterbodies (13.4 ± 2.7 TgCO_2_eq yr^−1^) stemmed from rivers (7.3 ± 1.2 TgCO_2_eq yr^−1^) and lakes (6.02 ± 1.5 TgCO_2_eq yr^−1^) and represented 9% of the total emissions, while waterbody relative area was 11% (Tables 1, 2). Emissions from wetlands (14.3 ± 1.8 TgCO_2_eq yr^−1^) included peat production sites (1.9 ± 0.4 TgCO_2_eq yr^−1^) and undrained mires (12.3 ± 1.8 TgCO_2_eq yr^−1^), representing 10% of both total area and total emissions. In the national inventory wetland emissions (2.2 TgCO_2_eq yr^−1^) consist mainly of emissions from peat extraction areas (Statistics Finland 2022a). The national inventory treats inland waters and undrained peatlands as unmanaged wetlands, and emissions are reported only for flooded land or land converted to inland waters.

The total C sequestration from the atmosphere to ecosystems was 93.2 ± 13.7 TgCO_2_eq yr^−1^, and thus the net emissions were 53.9 ± 15.3 TgCO_2_eq yr^−1^ (Table 2). Forest ecosystems on mineral soil was the main sink (NEE 79.9 ± 12.2 TgCO_2_eq yr^−1^), with additional sinks in forestry-drained peat soils (NEE 9.4 ± 6.1 TgCO_2_eq yr^−1^), and undrained peat soils (3.9 ± 0.4 TgCO_2_eq yr^−1^). Because of C sequestration the calculated GHG flux thus decreased by 63% from total emissions 147.2 ± 6.8 TgCO_2_eq yr^−1^.

Using the GWP_100_ metric, the main contribution (84%) to total emissions to the atmosphere was in the form of CO_2_ (123.4 TgCO_2_eq yr^−1^), while CH_4_ and N_2_O contributed 12% and 4% each (Table 2). As regards emissions from artificial surfaces and arable land, CO_2_ contributed 95% and 49% of total emissions. In forests, almost all (97%) of emissions came from CO_2_ calculated from C in harvested biomass, while CH_4_ and N_2_O from drained peatland soils gave rise to the remaining 1% and 2%, respectively. Evasion of CO_2_ from lake and river surfaces stood for 94% of the waterbody emissions, and CH_4_ from lakes for the remaining 6%. Wetland emissions amounted to 81% from CH_4_, 13% from CO_2_ and 7% from N_2_O.

**Fig. 2:**
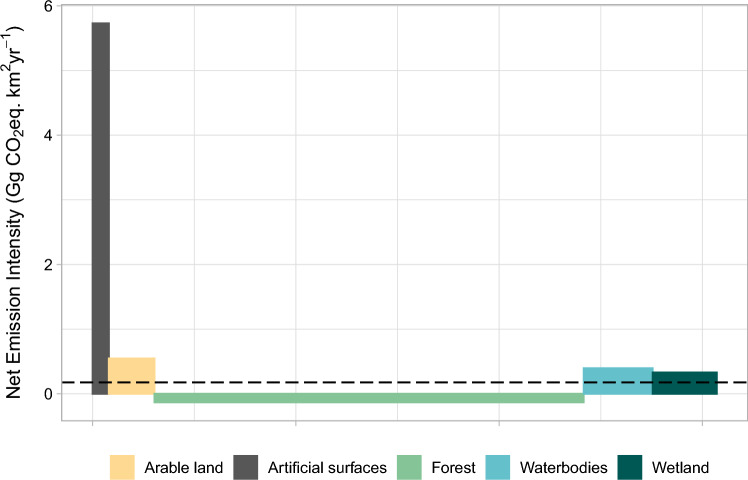
GHG emission intensity (GgCO_2_eq km^−2^ yr^−1^) versus relative area by land cover category in mainland Finland. Average emission intensity 0.18 GgCO_2_eq km^−2^ yr^−1^ (dashed line). Total net emissions to the atmosphere 53.9 TgCO_2_eq yr^−1^.

For all of Finland, artificial surfaces were by far the most emission intensive (5.7 GgCO_2_eq km^−2^ yr^−1^), followed by arable land (0.6 GgCO_2_eq km^−2^ yr^−1^), waterbodies (0.4 GgCO_2_eq km^−2^ yr^−1^), and wetland (0.3 GgCO_2_eq km^−2^ yr^−1^). Although losses of carbon from forests were higher than emissions from artificial surfaces, forests contributed the main sink, yielding a net emission intensity of -0.1 GgCO_2_eq km^−2^ yr^−1^. The country average net emission intensity was 0.2 GgCO_2_eq km^−2^ yr^−1^ (Table 2; Fig. 2).

Emissions from artificial surfaces clearly dominated regional emissions (78%) only in Uusimaa, which had the highest fraction of artificial surfaces and plants for energy production and industrial processing relative to country totals, 10% and 12%, respectively. In all other regions, artificial surfaces contributed to less than 50% of emissions. Carbon loss from forest harvest caused more than 60% of the emissions in four regions (South Savo, North Savo, North Karelia, and Central Finland). In all regions, carbon sequestration in forests dominated regional sequestration (> 87%), sequestration in undrained mires being the only other sink considered. In the south, and along the western coast, because of more favourable tree growth conditions, the sequestration intensity in forests was the highest (> 0.5 GgCO_2_eq km^−2^ yr^−1^). Despite lower temperatures, however, the extensive northern forests in Lapland and North Ostrobothnia secured 19% and 11% of the country total C sequestration in forests. In South and Central Ostrobothnia emissions from arable land represented 26% and 29% of the regions’ emissions, while Ostrobothnia and North Ostrobothnia received 13% and 14% of their total emissions from arable land. In all other regions, arable land emissions were 10% or less of total regional emissions. Lapland had the highest arable land emission intensity (1.4 GgCO_2_eq km^−2^ yr^−1^) because of its high fraction of cultivated organic soils (29% of the region’s agricultural land). On the country level, 86% of wetland emissions came from undrained mires, although peat production gave rise to more than 60% of wetland emissions in Satakunta, Pirkanmaa, Kymenlaakso, South Karelia, Central Finland and South Ostrobothnia. The highest net emission intensities from undrained mires were found in the northern regions with their large proportions of open and sparsely treed fens or sedge fens (Lapland, Kainuu, and North Ostrobothnia). Undrained mires provided C sequestration in wetlands, and the lowest emissions from undrained mires were in regions with large proportions of productive forested undrained mires and low proportion of sedge and other fens (Uusimaa, Päijät-Häme, South Karelia and South Savo).

**Fig. 3:**
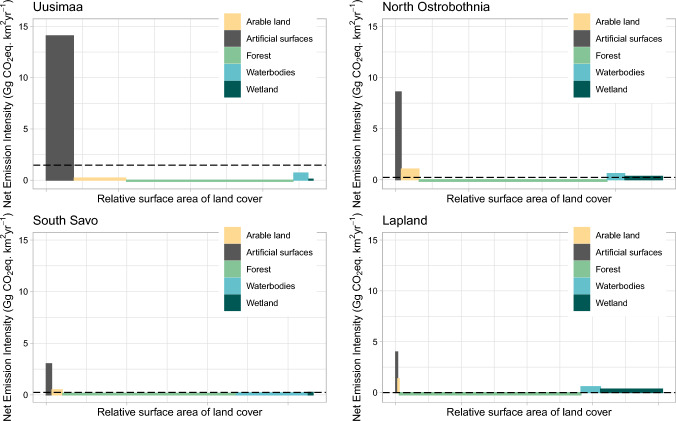
GHG net emission intensity (Gg CO_2_eq km^−2^ yr^−1^) versus relative area by land cover type in four contrasting regions. Average net emission intensity 1.40, 0.23, 0.21, and -0.001 Gg CO_2_eq km^−2^ yr^−1^ in Uusimaa, South Savo, North Ostrobothnia, and Lapland, respectively (dashed line). Region net emissions to the atmosphere 13.4, 4.0, 8.6, and -0.1 TgCO_2_eq yr^−1^ in Uusimaa, South Savo, North Ostrobothnia, and Lapland, respectively. The areas of the individual bars represent the net emissions for each land cover class

The net emission intensity of the five land cover classes was plotted for four contrasting regions Uusimaa, South Savo, North Ostrobothnia and Lapland (Fig. 3). The regions’ results are described briefly below, to illustrate the differences between the GHG fluxes in Finland.

**Fig. 4:**
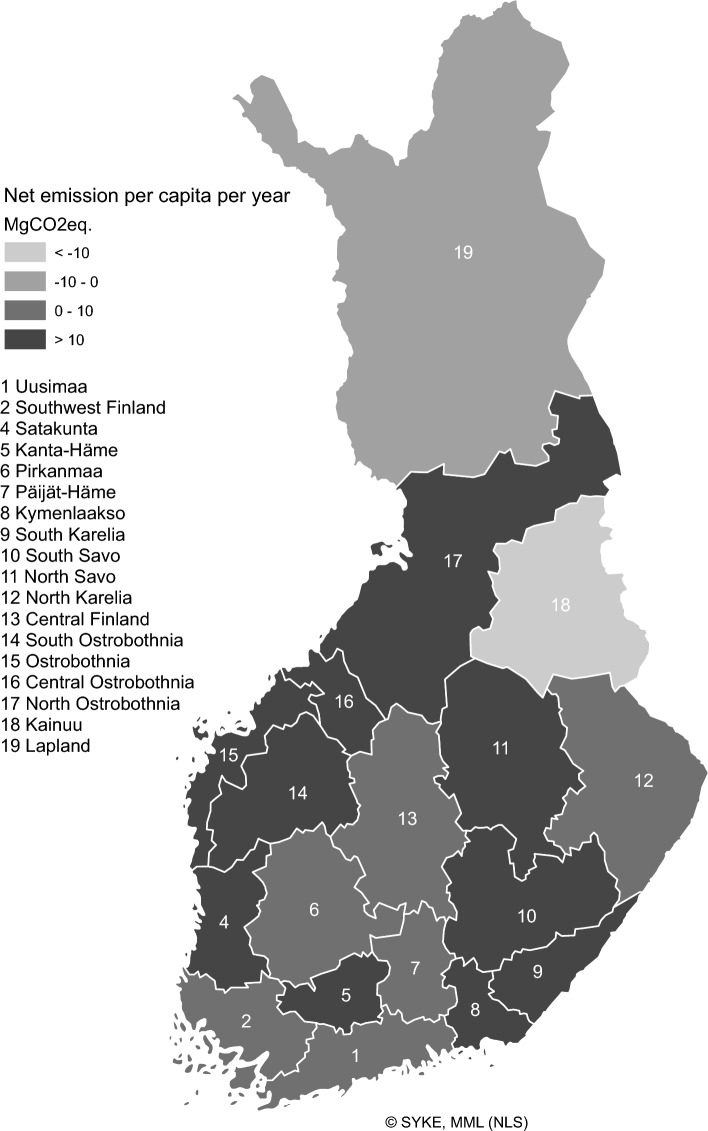
Net per capita emission (MgCO_2_eq yr^−1^) estimated for current conditions (2017 – 2025). Country average 9.8 MgCO_2_eq yr^−1^

Uusimaa (region 1) had the highest average net emission intensity (1.4 GgCO_2_eq km^−2^ yr^−1^) (Fig. 3), but its net per capita GHG 7.8 MgCO_2_eq yr^−1^ was lower than country average (Fig. 4; Table 4). Uusimaa is the most urbanized region (95.7%), with the highest population density (187 km^−2^), and the largest relative artificial surfaces area (10%) (Suppl. Tabs. S1, S17; Suppl. Figs. S1, S2, S4). This region had the country’s highest emissions from artificial surfaces, 29% of country total, major sources in Uusimaa being energy production, road traffic and industrial processes (Suppl. Tab. S12; Suppl. Fig. S5). Despite the relative arable land area being more than twice the country average (20%), most of the cultivated areas in Uusimaa were located on mineral soils, and therefore the relative role of arable land emissions was small (Suppl. Tab. S17; Suppl. Figs. S6, S7). Uusimaa relative forest area was lower (63%) than country average, but as the region’s forest harvest emissions were comparatively small, the relative role of the forest sink was above country average (Table S17).

**Table 4:** Summary of characteristics of regions in Fig. 3

Region	1 Uusimaa	10 South Savo	17 North Ostrobothnia	19 Lapland
Net emission intensity GgCO_2_eq/km^2^)	1.4	0.2	0.2	0.0
Net per capita emission MgCO_2_eq/yr	7.8	30.1	20.0	− 0.7
Degree of urbanization	96%	72%	84%	78%
Population density (1/km^2^)	187	10	11	2
Artificial surfaces area relative to region total area	11%	2%	2%	1%
Number of energy production and industrial processing plants relative to country total	12%	4%	9%	5%
Artificial surfaces emissions relative to region total emissions	79%	16%	43%	15%
Artificial surfaces area relative to country total artificial surfaces area	7%	3%	11%	2%
Artificial surfaces emissions relative to country total artificial surfaces emissions	29%	2%	14%	5%
Agricultural land area relative to region total area	20%	5%	7%	0.5%
Agricultural land area relative to country total agricultural land area	8%	3%	11%	2%
Cultivated organic soil area relative to total cultivated area in region	2%	7%	28%	29%
Agricultural emissions relative to region total emissions	2%	10%	14%	3%
Agricultural emissions relative to country total agricultural emissions	3%	3%	21%	5%
Forest area relative to region total area	65%	66%	71%	68%
Forest C loss in harvest relative to region total emissions	18%	71%	31%	21%
Forest C sequestration relative to region total C sequestration	100%	99%	94%	87%
Forest area relative to country total forest area	3%	5%	12%	26%
Forest C loss in harvest relative to country total emissions	5%	9%	9%	7%
Forest C sequestration relative to country total C sequestration	4%	5%	11%	19%
Waterbody area relative to region total area	5%	27%	7%	7%
Waterbody emissions relative to region total emissions	2%	11%	8%	18%
Waterbody area relative to country total waterbody area	1%	13%	7%	18%
Waterbody emissions relative to country total waterbody emissions	3%	7%	11%	26%
Wetland area relative to region total area	2%	2%	14%	20%
Wetland area of class 1 (Productive forested mires) relative to total wetland area	52%	61%	15%	6%
Wetland area of class 2 and 3 (Sedge fens and other open and sparsely treed fens) relative to total wetland area	12%	16%	66%	81%
Wetland emissions relative to region total emissions	0.2%	1%	13%	45%
Wetland C sequestration relative to region total C sequestration	1%	1%	6%	13%
Wetland area relative to country total wetland area	0.5%	0.9%	16%	62%
Wetland emissions relative to country total wetland emissions	0.2%	1%	17%	62%
Wetland C sequestration relative to country total C sequestration	0.4%	0.8%	16%	63%

South Savo (region 10), with 72% degree of urbanisation and population density of 10 km^−2^, had the highest net emission per capita (30 MgCO_2_eq yr^−1^). This is due to a combination of low population (2% of country total) and relatively high emissions (5% of country total), especially from forest harvest (9% of country total). Emissions from lakes were also high in South Savo (12% of country total lake emissions), because this region has the country’s highest proportion of inland waters (26% of total region area). South Savo’s share of sinks was 4% of country total, and the net emission intensity of South Savo (0.23 GgCO_2_eq km^−2^ yr^−1^) was slightly higher than the country average (0.16 GgCO_2_eq km^−2^ yr^−1^).

Domestic livestock production and cultivation of cropland in North Ostrobothnia (region 17) caused 21% of the country’s total arable land emissions. Although the area of cultivated organic soils was less than half of the cultivated mineral soils area, the organic soils caused 58% of the arable land emissions in this region. Emissions from artificial surfaces were also important in North Ostrobothnia (14% of country total), the share of industrial processes emissions being 42% of the corresponding country total, due to large industrial facilities in the region. The region’s degree of urbanisation was 84 and population density 11 km^−2^. Peat production in North Ostrobothnia represented 26% of country total, and forest emissions 9% of country total. The region’s total emissions were 13% and sinks 11% of the total of the 18 regions.

Lapland (region 19) had the lowest population density 2 km^−2^, and 78% degree of urbanisation. River emissions were 31% of total river emissions from all 18 regions. In Lapland, wetlands were the most important source of emissions, contributing 62% of country total. Undrained mires gave rise to 98% of Lapland’s wetlands emissions, with the remaining 2% from peat production. Sequestration on undrained mires was also important, 13% of Lapland’s total sink. Lapland had the highest sink in the country, representing 21% of country total. Lapland and Kainuu were the only two regions with zero or negative net emissions. Lapland’s and Kainuu’s net per capita emissions were -0.7 and -24.6 MgCO_2_eq yr^−1^, respectively.

## Discussion

The purpose of this study is to provide spatially explicit information on the emissions of all main land cover types in Finland, using methods that may also partly be applied to scenario analysis (Forsius et al. 2023). There are both similarities and differences between our approach and the national GHG inventory (Statistics Finland 2022a), both use the Yasso model to calculate forest mineral soil C balance, and we have, e.g. used the same area-based emission coefficients for cropland, field cultivation, and peat extraction emissions. The main differences between our approach and the national GHG inventory are related to how forest growth and litter input to forest soil are calculated. Instead of estimating the annual dynamics of forest biomass from statistics as in the GHG inventory, we used the dynamic forest growth model PREBAS, to enable scenario analysis, which is reported in accompanying papers by Forsius et al. (2023), Junttila et al. (2023) and Mäkelä et al. (2023). PREBAS used MS-NFI data (Mäkisara et al. 2022) as input, which compared to the NFI data used in the national GHG inventory is more centred at the mean values and underestimates small and large values, therefore the means are likely overestimated (and NEE could be too optimistic) (Haakana et al. 2022). The forest GHG emissions and C sequestration dominate the total balance, and the uncertainty of the forest C sink is the largest of the uncertainties. A similar result was reported by Monni et al. (2007). An omission on our part is that C storage in wood products was not included here, in contrast to the national inventory. To illustrate the fact that neutrality can be achieved either by decreasing the emissions or increasing the sequestration, we treat the emissions and sequestration separately (Table 2; Fig. 1). Another difference in the approach is that no emissions of inland waters or unmanaged wetlands are included in the official GHG reporting.

As our results indicate (Table 4; Fig. 3) there are large differences in the spatial distribution of the industrial emissions, the land use sector, peat extraction and surface waters and undrained mires between the different regions. This reflects the uneven distribution of population, industrial activities, peat areas, and land use management. Similarly, the regional distribution of high emission sources from arable land on organic soils is very uneven, with highest proportion in western and northern regions (Regina et al. 2019). The updated national climate law has ambitious goals for GHG emission reductions and increasing the net sink of the LULUCF sector. There is, however, currently no clear integrated national policy to steer these developments and to assist the regions to reach their regional targets. Our spatially explicit datasets aim at assisting both regional land use planning and provide information for national considerations.

The uncertainties related to the sink estimates were higher than those of the emissions. On the emission side, the largest uncertainties were related to the agriculture sector and drained forested peat soils. Our uncertainty estimates were calculated from the uncertainties in emission coefficients and Monte Carlo analysis of the PREBAS simulations (Junttila et al. 2023). Additional uncertainty that we did not address is related to the areal data sources and the spatial allocation of emission sources. Furthermore, we used static coefficients for the GHG balance of peat soils, reported from long-term studies, although emissions are likely to vary with climate. Uncertainties in FRES modelling of artificial surfaces emissions are, e.g. spatial proxies for residential wood combustion (Paunu et al. (2021). Junttila et al. (2023) provide a detailed evaluation of uncertainties related to PREBAS-based scenario modelling, such as the current state of the forest, model parameters, and climate models, concluding that multiple modelling approaches with uncertainty estimates are needed to inform policy planning. The major uncertainty involved in large-scale quantification of GHG emissions and sinks is not widely recognized, even though decisions are made on regulations involving both long time frames and having substantial financial implications. Communication on both quantitative GHG information and their uncertainties is thus a key task.

Our results on current GHG emissions from 18 regions in mainland Finland, and the relative importance of different land cover classes for the net emissions of each region introduces additional information with regards to the national GHG inventory (Statistics Finland 2022a). Because our data is based on municipal sums and gridded information, the results can be used also at smaller scales (e.g. individual municipalities), however accounting for the increasing uncertainty concerning smaller spatial units presents challenges. Many regions and municipalities have action plans for implementing regional and local emission reductions and climate roadmaps (Saikku et al. 2022). Some regions plan to subdivide the regions into different spatial units for regional development, with focus on activities such as recreation, intensive land use and industrial activities. Our results aim at supporting these activities and we will provide documented data products to the regional actors to provide data support for these plans. Detailed analysis of the regional mitigation potential is, however, beyond the scope of this work (Bianchi et al. 2021; Roe et al. 2019). Climate mitigation efforts may be combined with biodiversity conservation schemes (Kangas and Ollikainen 2022; 2023). Shin et al. (2022) argued for better integration of biodiversity conservation and climate change mitigation into management and policy. Smith et al. (2023) showed that it is possible to meet climate and biodiversity targets, but policies must be designed carefully to manage trade-offs and deliver multiple sustainability objectives. Multiple sources of data (NFI, remote sensing, UAV) can be integrated in our modelling framework, with the potential of increasing the accuracy of land cover data and the quantification of actual forest state (Miettinen et al. 2021), which would improve C monitoring.

## Conclusion

Regional and local decisions are necessary for implementing national and global targets of climate mitigation. Spatially explicit information on the relative importance of different land cover forms on net emissions of greenhouse gases (GHG) and carbon stocks is needed to inform such actions. We illustrated the differences in 18 regions in mainland Finland by calculating detailed emission and sequestration balances for land cover forms of artificial surfaces, cropland, forests, waterbodies, and wetland. Our results show large regional contrasts that reflect both long-term economic developments and natural factors. On the country level, the role of forest (carbon losses in timber and energy wood harvest and soil emissions from drained peatland) amounted to 42% of total country emissions. Artificial surfaces (energy production, industrial processes, road traffic, agriculture, machinery and off-road transport, waste management, peat production, residential combustion), caused 31% of total emissions. Forests also provided the main sink, 96% of total sequestration. Our results aim at supporting implementation of regional climate roadmaps and sustainable land use, and thereby assist reaching also national targets. There are still large uncertainties in the spatial GHG information that need further work, e.g. regarding the current state of the forest, and proxies for distributing emissions from artificial surfaces. Regional and national decision-making would benefit from multiple modelling approaches including uncertainty estimates of GHG emissions and C sinks.

Data: Regional greenhouse gas net emission intensities by land cover category in Finland https://doi.org/10.5281/zenodo.7827577

### Supplementary Information

Below is the link to the electronic supplementary material.

Supplementary file1 (PDF 887 kb)
