# Supplementary material for: Role of land cover in Finland’s greenhouse gas emissions
Source: Ambio. 2023 Sep 7;52(11):1697–715. doi: 10.1007/s13280-023-01910-8 (PMC10562319; doi:10.1007/s13280-023-01910-8)
Supplement: Supplementary file 1 — Supplementary file1 (PDF 887 kb) [file 13280_2023_1910_MOESM1_ESM.pdf]

Ambio

Supplementary information

This supplementary information has not been peer reviewed.

Title: **Role of land use in national greenhouse gas emissions**

Authors: Maria Holmberg, Virpi Junttila, Torsti Schulz, Juha Grönroos, Ville-Veikko Paunu, Mikko Savolahti, Francesco Minunno, Paavo Ojanen, Anu Akujärvi, Niko Karvosenoja, Pirkko Kortelainen, Annikki Mäkelä, Mikko Peltoniemi, Jouko Petäjä, Pekka Vanhala, Martin Forsius

Contents

Supplementary information ... 1

Summary Information on ALas ... 3

Summary of PREBAS model runs ... 4

Examples of regional differences ... 4

Figures ... 6

Figure S1. Degree of urbanization (%) ... 6

Figure S2. Population density (km-2) ... 6

Figure S3. Net emission intensity (GgCO2eq km^{-2} yr^{-1}). ... 6

Figure S4. Relative area of artificial surfaces. ... 7

Figure S5. Relative emissions of artificial surfaces. ... 7

Figure S6. Relative area of arable land. ... 7

Figure S7. Relative emissions of arable land ... 7

Figure S8. Relative area of forests. ... 8

Figure S9. Relative emissions of forests. ... 8

Tables ... 9

Table S1. Administrative regions ... 9

Table S2. Compound molar masses and greenhouse gas warming potentials (GWP, AR5 GWP (100)). ... 10

Table S3 Emission flux rates from peat production sites, given as mean (standard deviation) ... 10

Table S4. Spatially explicit data sources ... 11

Table S5. Source- and GHG-level uncertainties of calculated emissions ... 13

Table S6. Emission flux rates from arable land, given as mean (standard deviation) ... 14

Table S7. Carbon flux rates of lakes for five lake size classes, given as mean (standard deviation), standard deviations as in Vanhala et al. (2016). ... 15

Table S8. Methane flux rates for different macrophytes, given as mean (standard deviation), standard deviations as in Vanhala et al. (2016). ... 16

Table S9. Carbon flux rates of rivers of different size classes, given as mean (standard deviation), standard deviations as in Vanhala et al. (2016). ... 16

Table S10 Classification of mire types based on multisource national forest inventory site classification. ... 17

Table S11a Emission flux rates of undrained mires, given as mean (standard deviation) ... 18

Table S11b Emission flux rates of drained forested peatland, given as mean (standard deviation) (Junttila et al. 2022) ... 18

Table S12. Artificial surfaces area (km^2) and emissions by region for 2019 ($TgCO_2eq$ yr^{-1}) ... 19

Table S13 Arable land area (km^2) and emissions by region ($TgCO_2eq$ yr^{-1}) ... 20

Table S14 Forest area (km^2). emissions and sequestration by region ($TgCO_2eq$ yr^{-1}) ... 21

Table S15 Waterbody area (km^2) and emissions by region for ($TgCO_2eq$ yr^{-1}) ... 23

Table S16 Undrained mires area (km^2) and emissions by region for ($TgCO_2eq$ yr^{-1}) ... 24

Table S17 Relative area (km^2/km^2) and relative emission ($TgCO_2eq/TgCO_2eq$) by region for each land cover class. ... 25

References ... 26

Summary Information on ALas

The ALas model is a tool for regional GHG calculation for Finnish municipalities with information for the years 2015 – 2019 (Lounasheimo et al. 2020).

Waste management (from Lounasheimo et al. 4.12 Jätteiden käsittely)

Emissions from waste management consist of CH4 from dump sites, CH4 and N2O from wastewater treatment, and CH4 and N2O from biological treatment, i.e., composting and decomposition. Emissions from dump sites are calculated with the First Order of Decay method (IPCC 2006). Information on the annual amount of waste dumped, its composition and decomposition properties, and the recovery of waste gases is input to the calculations. Default parameters used in the national GHG inventory are applied (Statistics Finland 2022), with information on waste amount and gas recovery on the municipality and dump site level. For emissions from biological and wastewater treatment, National GHG inventory values are allocated to municipalities based on information on population and the building area of industrial plants. For each municipality, waste management emissions are reported for each municipality according to the amount of waste produced in each municipality, regardless of where the waste was managed, and aggregated to the region in which the municipality is located.

Agriculture (from Lounasheimo et al. 4.11 Maatalous)

Emissions from agriculture consist of CH_4 and N_2O from domestic livestock production (enteric fermentation and manure management), and CO_2 from field cultivation (liming and urea application) (Table ALas 11). Information on the number of livestock in each municipality is input to the calculations. Emissions from field cultivation use information on the cultivated area of different crops in each municipality and the crop yield in each region, as well as national level usage of agricultural liming material, urea, mineral nitrogen fertilizer and municipal sewage sludge.

Supplementary Table ALas 11

CRF*	ALas-Classification	Emission source	CO_2	CH_4	N_2O
3.A	Enteric fermentation	Domestic enteric fermentation		X	
3.B	Manure management	Manure management		X	X**
3.F	Field Cultivation, other emissions	Field Burning of Agricultural Resiudes		X	X
3.G	Field Cultivation, other emissions	Liming	X		
3.H	Field Cultivation, other emissions	Urea Application	X		
3.D.a		Direct Soil Emissions			X
	Inorganic Fertilizers	Syntetic Fertilizers			X
	Organic Fertilizers	Animal Manure Applied to Soils			X
	Organic Fertilizers	Municipal Sewage Sludge Applied to Soils			X

	Organic Fertilizers	Domestic Livestock Grazing			X
	Field Cultivation, other emissions	Crop Residue			X
	Soil	Mineralisation associated with loss of soil organic matter (mineral soils)			X
	Soil	Cultivation of Histosols			X
3.D.b		Indirect N_2O Emissions			X
	Field Cultivation, other emissions	Atmospheric Deposition			X
	Field Cultivation, other emissions	Nitrogen Leaching and Run-off			X

*CRF (Common Reporting Format) UNFCC
** Direct and Indirect N_2O emissions

Summary of PREBAS model runs

Carbon sequestration in forest biomass was simulated with PREBAS, which is a dynamic process-based model of carbon assimilation and tree growth (Minunno et al. 2016, 2019). PREBAS is initialized using forest structural variables (i.e. average height of the stand, average diameter at breast height, basal area). The model is initialized for the three main species in Finland: Scots pine, Norway spruce and Silver birch. Information on the initial state of the forest is based on data from the multi-source national forest inventories (MS-NFI), that provide forest variables at 16 m resolution. Regional harvesting intensities are modelled on the basis of the Finnish national statistics and consist of annual levels of roundwood, pulpwood and energy wood.

Examples of regional differences

Cropland cultivation on mineral soil in Southwest Finland (region 2) contributed 15% of total mineral soil emissions from cropland in the 18 regions. Cropland cultivation on mineral soil was an important source of emissions in Satakunta (region 4) (9% of country total mineral soil cropland GHG). Peat as fuel in energy production caused also high emissions in Satakunta (15% of country total energy production with peat). Total anthropogenic emissions in Satakunta represented 5% of the country total, while forest emissions were 4% of country total. Satakunta total sink was 3% of the total sink of mainland Finland.

Average intensities of 0.4 $GgCO_2eq\ km^{-2}\ yr^{-1}$ or higher occurred in Satakunta, Kanta-Häme and South Karelia (regions 4, 5, and 9). Southwest Finland, Pirkanmaa, Päijät-Häme (2, 6, 7), all four Ostrobothnian regions (14, 15, 16, 17), and both Savo regions (10, 11) landed on average intensities of 0.2 or 0.3 $GgCO_2eq\ km^{-2}\ yr^{-1}$. Kainuu (region 18), Lapland (19), North Karelia and Central Finland (12, 13) had intensities of -0.1 to 0.1 $GgCO_2eq\ km^{-2}\ yr^{-1}$ (Table 4).

South Savo (region 10) had the highest net emission per capita 30 $MgCO_2eq\ yr^{-1}$. South Karelia and North Ostrobothnia (regions 9 and 17) had also per capita GHG > 20 $MgCO_2eq\ yr^{-1}$. Only Kainuu and Lapland (regions 18 and 19) had negative per capita emission. The GHG per capita in the remaining 13 regions were between 6 and 15 $MgCO_2eq\ yr^{-1}$ (Table 4).

In all of Ostrobothnia (regions 14, 15, 16, 17), as well as in Satakunta and Kanta-Häme (regions 4, 5) the relative importance of cropland emissions was higher than the country average (5%) (Suppl. Fig. S7). In Central and North Ostrobothnia, Kainuu and Lapland (regions 16, 17, 18, 19) more than a quarter of the cultivated area was on organic soils. For these regions 98% of the cropland emissions were from organic soils. For the whole country the importance of the organic soils for cropland emissions was almost equally high, 93% (Suppl. Tab. S13).

Forests on mineral soils caused on average 81% of the total forest emissions. Only in North Karelia, South, Central and North Ostrobothnia, Kainuu and Lapland (regions 12, 14, 16, 17, 18, 19) the role of forested mineral soils was lower than 20% (Suppl. Tab. S14). The carbon sequestration in forests on mineral soil represented 89% of the total forest sink on the country level. Only in South, Central and North Ostro-bothnia and in Kainuu did less than 90% of the total forest sink occur on upland forest soils.

Figures

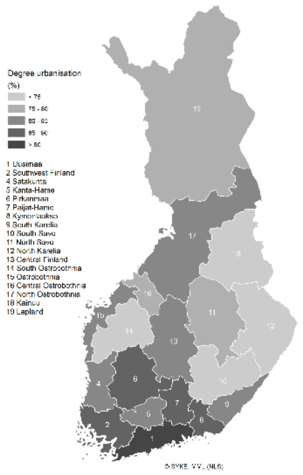

Figure S1. Degree of urbanization (%) of 18 administrative regions of mainland Finland (National Land Survey of Finland, 2022).

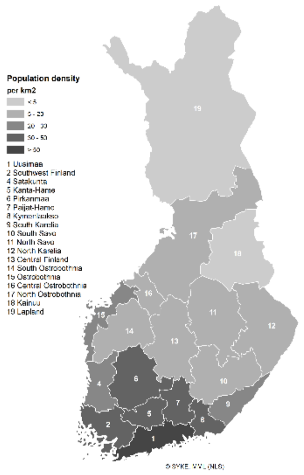

Figure S2. Population density (km-2) of 18 administrative regions in mainland Finland (National Land Suvey Finland 2022; Statistics Finland 2022a).

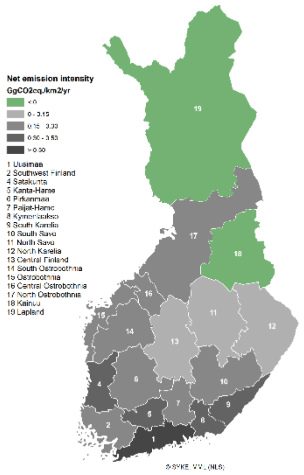

Figure S3. Net emission intensity (GgCO2eq km^{-2} yr^{-1}). Regional averages for 18 administrative regions of mainland Finland.

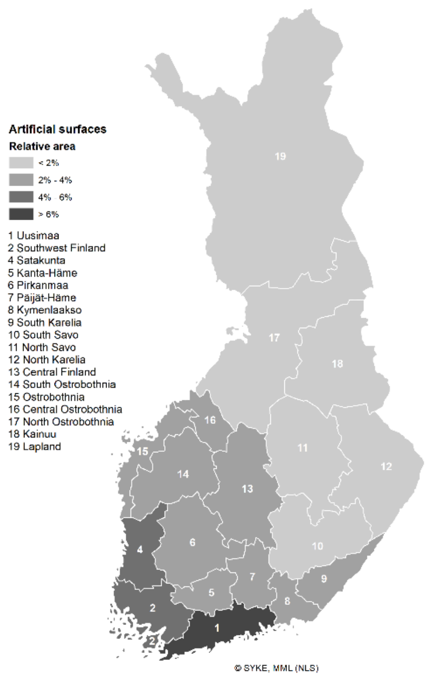

Figure S4. Relative area of artificial surfaces.

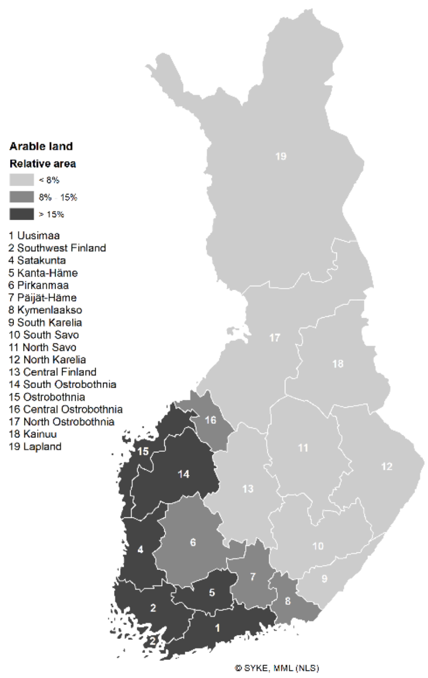

Figure S6. Relative area of arable land.

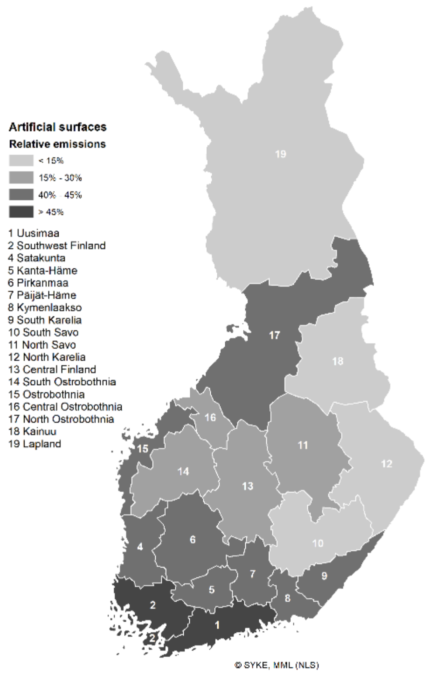

Figure S5. Relative emissions of artificial surfaces.

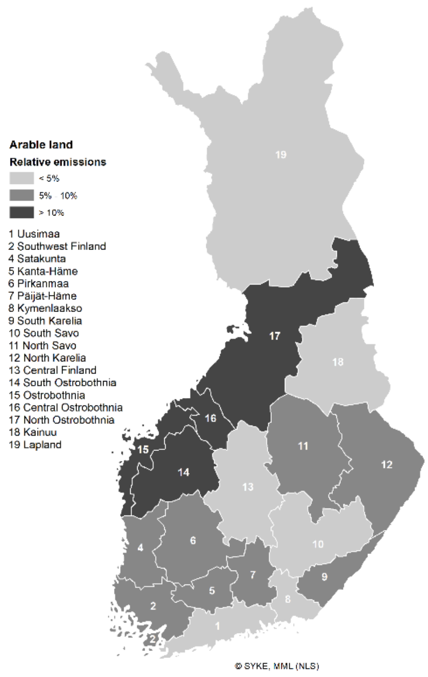

Figure S7. Relative emissions of arable land.

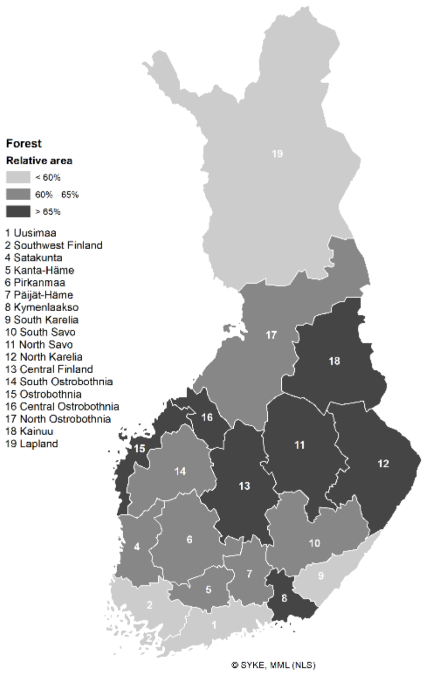

Figure S8. Relative area of forests.

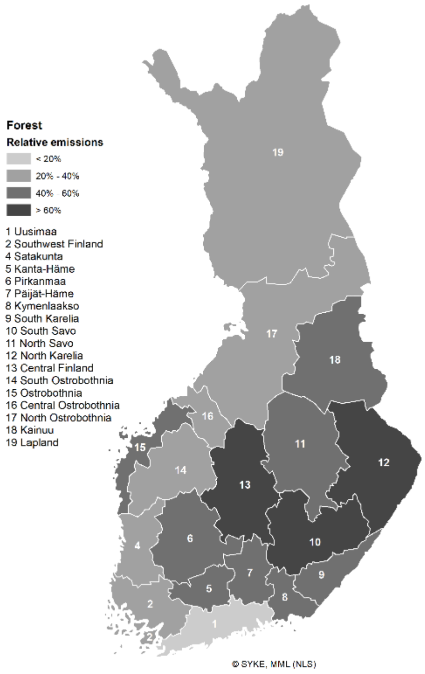

Figure S9. Relative emissions of forests.

Tables

Table S1. Administrative regions

Region code [1] (NUTS 3)	Region code (FI)	Region name	Land area[2] (km^2)	Inland water area[2] (km^2)[2]	Area including land and inland[2] water (km^2)	Population[3] 31.12.2020	Population density (km^{-2})	Degree of urbanisation[3] (%)
FI1B1	1	Uusimaa	9 099	470	9 569	1702678	187	95.7
FI1C1	2	Southwest Finland	10 668	246	10 914	481403	45	85.9
FI196	4	Satakunta	7 823	447	8 269	215416	28	83.6
FI1C2	5	Kanta-Häme	5 199	508	5 708	170577	33	83.0
FI197	6	Pirkanmaa	13 249	2 300	15 550	522852	39	88.7
FI1C3	7	Päijät-Häme	5 714	1 228	6 942	205771	36	87.5
FI1C4	8	Kymenlaakso	4 559	389	4 948	162812	36	88.4
FI1C5	9	South Karelia	5 326	1 546	6 872	126921	24	84.3
FI1D1	10	South Savo	12 652	4 447	17 099	132702	10	72.2
FI1D2	11	North Savo	17 345	3 733	21 078	248265	14	76.6
FI1D3	12	North Karelia	18 793	4 110	22 903	163537	9	73.0
FI193	13	Central Finland	16 042	2 970	19 012	272617	17	81.5
FI194	14	South Ostrobothnia	13 798	557	14 356	192150	14	73.8
FI195	15	Ostrobothnia	7 401	178	7 580	175816	24	84.7
FI1D5	16	Central Ostrobothnia	5 020	204	5 224	67988	14	80.0
FI1D6	17	North Ostrobothnia	36 830	2 364	39 194	413830	11	84.2
FI1D4	18	Kainuu	20 198	2 490	22 688	71664	4	74.7
FI1D7	19	Lapland	92 678	6 304	98 982	176665	2	78.2
		Mainland Finland	302 394	34 493	336 887	5503664	18	86.7

[1]International NUTS classification. Available at: https://www.stat.fi/en/luokitukset/nuts/. Accessed 14.6.2023
[2]National Land Survey of Finland, 2022.
[3]Statistics Finland, 2022.

Table S2. Compound molar masses and greenhouse gas warming potentials (GWP, AR5 GWP (100)).

Compound	Global warming potential (GWP)*
Carbon dioxide CO_2	1
Methane CH_4	28
Nitrous oxide N_2O	265

*) GWP values for 100-year time horizon AR5 (IPCC 2014, Myhre et al. 2013, p. 714)

Table S3 Emission flux rates from peat production sites, given as mean (standard deviation)

Description	Unit	Mean (std)	Reference
CH_4 flux	g CO2eq m^{-2} yr^{-1}	46.8 (9.4)	Nykänen et al. 1996, Alm et al. 2007
CO_2 flux	g CO2eq m^{-2} yr^{-1}	1461.5 (292.3)	Nykänen et al. 1996, Alm et al. 2007
N_2O flux	g CO2eq m^{-2} yr^{-1}	89.5 (17.9)	Nykänen et al. 1996, Alm et al. 2007

Table S4. Spatially explicit data sources

Land use	Temporal representation	Spatial resolution	Data sources	Used models	Sectors/ Processes	Links to metadata on spatial data sources
Artificial surfaces	2019 (energy production and industrial plants as several year averages)	Point sources, and area sources with resolution 250 m x 250 m	Digiroad for roads and traffic volumes, The National Buildings and Dwellings Register for buildings data, and CORINE2012 and 2018 for land use data. Level 2 land cover classes 11, 12 and 13	FRES, Karvosenoja 2008, Paunu et al 2013, Karvosenoja et al 2018: CO_2, CH_4, N_2O -> CO2eq.	Industrial processes (CO_2); Energy production (CO_2);<br>Road traffic (CO_2);<br>Machinery and off-road transport (CO_2);<br>Waste management (CH_4, N_2O);<br>Residential and other small-scale combustion (CH_4, CO_2, N_2O);	CLC2018:https://ckan.ymparisto.fi/dataset/%7B0B4B2FAC-ADF1-43A1-A829-70F02BF0C0E5%7D
Arable land	2020	Polygons	Land Parcel Information System 2020; Mineral/organic: the soilscapes of the national digital soil map.	Min/org classification: Lilja et al (2006, 2017)	Agriculture (CH_4, CO_2, N_2O);<br><br>Area-specific coefficients: CO_2, N_2O	DOI 10.5281/zenodo.7827577
Forest	2017-2025	16 m x 16 m to homogeneous units	MS-NFI. NFI field measurements, satellite imagery and digital map data (Tomppo et al. 2014). Forest data was further segmented to homogeneous units.	Forest growth and gas exchange: PREBAS (Minunno et al. 2016, 2019) with forest management actions in accordance with current best practices (Sved and Koistinen 2015).	Soil respiration: Mineral(upland) soils soil carbon model YASSO07 (Tuomi et al. 2009). Drained peatland(organic) soils: measured soil respiration including both peat decomposition and litter decomposition (or accumulation) (Minkkinen et al. 2018; Ojanen et al. 2010, 2013, 2019)	DOI 10.5281/zenodo.7827577

Waterbodies	2021	Lake polygons -> Lake surface area<br>River polygons (width >= 5m)/<br>River lines (width < 5 m; average width assumed = 3.5 m)	River network data set by SYKE	Kortelainen et al. (2006), Bastviken et al. (2004), Juutinen et al. (2009), Haaspuro (2013): CO_2, CH_4-> CO2eq.<br>Humborg et al. 2010, Haaspuro 2013: CO2eq.	Lakes: Area-specific coefficients: CO_2 evasion, CH_4 diffusion, CH_4 ebullition, C accumulation<br>Rivers:<br>Area-specific coefficients: CO_2 evasion	https://ckan.ymparisto.fi/dataset/%7BE15CD0B3-3934-4ABC-BF23-A8C622FA6A57%7D<br><br>DOI 10.5281/zenodo.7827577
Wetland	2019	Spatial shapefile -> Undrained peatland area.	National drained peatland mask by Luke. Forest soil and type classification from MS-NFI 2019 (Mäkisara et al. 2022).	Area based, CO_2, CH_4, N_2O calculated with emissions factors -> CO2eq	Peat production (CH_4, CO_2, N_2O);<br><br>Area-specific coefficients: CO_2, CH_4, N_2O emission, C accumulation. Minkkinen and Ojanen 2013, Turunen et al. 2002, Sallantaus 1994, Minkkinen et al. 2020.	DOI 10.5281/zenodo.7827577

Table S5. Source- and GHG-level uncertainties of calculated emissions

		CH_4 ($GgCO_2$-eq yr^{-1})	CO_2 ($GgCO_2$-eq yr^{-1})	N_2O ($GgCO_2$-eq yr^{-1})
Energy production and industrial processes	Liquid fuels	±36%	±4%	-40/+41%
	Waste and other solid		±2%	
	Gaseous fuels		±1%	
	Peat combustion	±60%	±3%	±60%
	Biomass combustion	±56%		-53/+54%
Area sources	Residential and other small scale combustion	-70/+152%	±6%	-72/+163%
	Road traffic		±3%	
	Machinery and off-road transport		±6%	
	Agriculture	-14/+18%		-37/+124%
	Peat production	±20%	±20%	±20%
	Waste management	-34/+33%		-59/+85%

Table S6. Emission flux rates from arable land, given as mean (standard deviation)

		Type of soil;Type of crop				Reference
		Mineral soil, South; All crops	Mineral soil, North; All crops	Organic soil; Annual crops	Organic soil, Perennial crops	
Description	Unit	Mean (std)	Mean (std)	Mean (std)	Mean (std)	
CO_2 emission	tC ha^{-1} yr^{-1}	0.073 (0.038)	0.047 (0.018)	7.9 (0.7)	5.7 (1.5)	Statistics Finland, 2023, Table 3_App_6j; IPCC 2014, Table 2.1
N_2O emission	kg N_2O-N ha^{-1} yr^{-1}	-	-	13.0 (2.5)	9.5 (2.4)	Statistics Finland, 2023, Table 5.4-8; IPCC 2014, Table 2.5

Table S7. Carbon flux rates of lakes for five lake size classes, given as mean (standard deviation), standard deviations as in Vanhala et al. (2016).

		Lake size class					
		1	2	3	4	5	
Description	Unit	0.01 – 0.1 km^2	0.1 – 1 km^2	1 – 10 km^2	10 – 100 km^2	> 100 km^2	Reference
CO_2 Evasion	gC m^{-2} yr^{-1}	102 (20.4)	66 (13.2)	56 (11.2)	37 (7.4)	25 (5.0)	Kortelainen et al. 2006, Table 4
CH_4 Diffusion	gC m^{-2} yr^{-1}	0.984 (0.20)	0.419 (0.08)	0.208 (0.04)	0.105 (0.02)	0.105 (0.02)	Haaspuro 2013; Juutinen, unpublished calculations based on data in Juutinen et al. 2009
CH_4 Ebullition	gC m^{-2} yr^{-1}	0.614 (0.12)	0.424 (0.08)	0.290 (0.06)	0.207 (0.04)	0.158 (0.03)	Bastviken et al. 2004*
Emergent macrophyte coverage	fraction of lake area	0.044 (0.010)	0.053 (0.010)	0.037 (0.007)	0.017 (0.003)	0.006 (0.001)	Haaspuro 2013; Calculated with Eq. 2 in Table 3 of Bergström et al. 2007

*Bastviken et al. (2004, Table 2, Equation: log(ebullition per lake) = 1.190 + 0.841 log(lake area). This equation was used by Haaspuro (2013) to derive average ebullition coefficients for each lake size class.

Table S8. Methane flux rates for different macrophytes, given as mean (standard deviation), standard deviations as in Vanhala et al. (2016).

		Macrophyte species		
Description	Unit	*Phragmites australis*	*Equisetum fluviatile*	Reference
Species distribution		0.37 (0.07)	0.45 (0.09)	Bergström et al. 2007
CH_4 evasion	$gCH_4\ m^{-2}\ yr^{-1}$	60.74 (12.15)	15.28 (3.06)	Haaspuro 2013; Juutinen, unpublished calculations based on data in Juutinen et al. 2003

Table S9. Carbon flux rates of rivers of different size classes, given as mean (standard deviation), standard deviations as in Vanhala et al. (2016).

		River width size class (m)				
		1	2	3	4	
Description	Unit	< 5 m	5 – 10 m	10 – 30 m	> 30 m	Reference
CO_2 evasion	$gC\ m^{-2}\ yr^{-1}$	2834 (566.8)	1786 (357.2)	1054 (210.8)	473 (94.6)	Humborg et al. 2010, Tables 7 and 1

Table S10 Classification of mire types based on multisource national forest inventory site classification.

	Mire class			
MSNFI 2019 classification[1]	1 Productive forested mires	2 Sedge fens	3 Other open and sparsely treed fens	4 Ombrotrophic bogs
Site main class	2 & 3	4	3; 4; 2; 3; 4	3
Site fertility class	1 - 4	3	1-3; 1-2; 1-4; 4; 4-6	5&6
Land class	1		2 & 3	
Total area (km^2)	3 851	6 599	14 362	5 696

[1]MS-NFI Classification Mäkisara et al. 2022

Table S11a Emission flux rates of undrained mires, given as mean (standard deviation)

		Mire class				
		1 Productive forested mires	2 Sedge fens	3 Other open and sparsely treed fens	4 Ombrotrophic bogs	
Description	Unit	Mean (std)	Mean (std)	Mean (std)	Mean (std)	Reference
CH_4 emission	$gCH_4\ m^{-2}\ yr^{-1}$	2.0 (3.4)	24.0 (2.0)	15.0 (1.6)	5.0 (1.9)	Minkkinen and Ojanen 2013
CO_2 net emission	$gCO_2\ m^{-2}\ yr^{-1}$	-116 (18)	-148 (12)	-126 (14)	-119 (12)	Turunen et al. 2002, Sallantaus 1994
N_2O emission	$gN_2O\ m^{-2}\ yr^{-1}$	0.11 (0.01)	0.11 (0.01)	0.11 (0.01)	0.08 (0.03)	Minkkinen et al. 2020

Table S11b Emission flux rates of drained forested peatland, given as mean (standard deviation) (Junttila et al. 2022)

		Site type		
		Nutrient rich sites (herb-rich type, blueberry type)	Nutrient poor sites (lingonberry type, dwarf-shrub type and lichen type	
Description	Unit	Mean (std)	Mean (std)	Reference
CH_4 emission	$gCH_4\ m^{-2}\ yr^{-1}$	0.34 (0.12)	0.34 (0.12)	Ojanen et al. 2010
CO_2 net emission	$gCO_2\ m^{-2}\ yr^{-1}$	240 (70)	-70 (30)	Ojanen and Minkkinen 2019
N_2O emission	$gN_2O\ m^{-2}\ yr^{-1}$	0.23 (0.04)	0.077 (0.004)	Minkkinen et al. 2020

Table S12. Artificial surfaces area (km^2) and emissions by region for 2019 ($TgCO_2eq\ yr^{-1}$)

Reg. Code	Region	Artificial surfaces area[1] (km^2)	Industrial Processes	Energy production - Waste and Other solids	Energy production - Peat	Energy production - Gaseous Fuels	Energy Production Liquid Fuels	Energy production - Biomass	Road Traffic	Machinery and Off-road Transport	Waste Manage-ment	Residential and other small-scale combustion	Artificial surfaces emissions, total ($TgCO_2eq\ yr^{-1}$)	Artificial surfaces emissions, relative to country total (%)
1	Uusimaa	941	2.026	3.832	0.352	2.832	0.565	0.003	2.304	0.730	0.310	0.295	13.248	29 %
2	Southwest Finland	575	0.822	0.781	0.126	0.096	0.072	0.004	0.862	0.280	0.117	0.189	3.350	7 %
4	Satakunta	460	0.202	0.250	0.884	0.128	0.082	0.005	0.444	0.135	0.102	0.131	2.362	5 %
5	Kanta-Häme	243	0.075	0.097	0.187	0.244	0.121	0.001	0.425	0.077	0.052	0.066	1.346	3 %
6	Pirkanmaa	604	0.030	0.062	0.590	0.489	0.065	0.004	0.965	0.183	0.183	0.179	2.751	6 %
7	Päijät-Häme	296	0.008	0.177	0.161	0.123	0.088	0.002	0.469	0.084	0.106	0.069	1.286	3 %
8	Kymenlaakso	215	0.152	0.085	0.026	0.388	0.078	0.008	0.350	0.081	0.157	0.085	1.409	3 %
9	South Karelia	299	0.572	0.055	0.102	0.242	0.088	0.012	0.289	0.057	0.186	0.059	1.661	4 %
10	South Savo	334	0.088	0.012	0.249	0.072	0.053	0.003	0.347	0.081	0.057	0.049	1.010	2 %
11	North Savo	520	0.016	0.079	0.466	0.089	0.072	0.006	0.523	0.155	0.093	0.074	1.572	3 %
12	North Karelia	434	0.039	0.017	0.215	0.113	0.083	0.004	0.346	0.121	0.062	0.051	1.051	2 %
13	Central Finland	483	0.000	0.012	0.422	0.075	0.056	0.015	0.615	0.131	0.091	0.105	1.521	3 %
14	South Ostrobothnia	491	0.010	0.015	0.532	0.023	0.017	0.001	0.452	0.136	0.068	0.129	1.383	3 %
15	Ostrobothnia	259	0.009	0.936	0.045	0.041	0.040	0.011	0.329	0.188	0.052	0.101	1.751	4 %
16	Central Ostrobothnia	183	0.019	0.001	0.205	0.007	0.005	0.001	0.135	0.048	0.026	0.041	0.489	1 %
17	North Ostrobothnia	759	3.533	0.061	0.752	0.590	0.096	0.010	0.877	0.297	0.163	0.144	6.521	14 %
18	Kainuu	271	0.000	0.005	0.138	0.032	0.035	0.003	0.172	0.099	0.031	0.027	0.541	1 %
19	Lapland	606	0.909	0.009	0.350	0.139	0.049	0.008	0.469	0.281	0.125	0.088	2.427	5 %
	Total	7 973	8.511	6.485	5.801	5.721	1.666	0.100	10.373	3.164	1.979	1.882	45.682	100 %

[1] Sum land cover area for Corine2018 Level 2 classes 11, 12 and 13

Table S13 Arable land area (km^2) and emissions by region ($TgCO_2eq$ yr^{-1})

Region Code	Region	Agricultural land[1] (km^2)	Cultivated land[2] (km^2)	Mineral soil area (km2)	Organic soil area (km2)	Domestic livestock production (TgCO2eq/yr)	Field cultivation (TgCO2eq/yr)	Cropland emissions mineral soils (TgCO2eq/yr)	Cropland emissions organic soils, (TgCO2eq/yr)	Arable land emissions total (TgCO2eq/yr)
1	Uusimaa	1 782	1 564	1 531	33	0.08	0.17	0.04	0.09	0.37
2	Southwest Finland	2 912	2 611	2 565	46	0.16	0.29	0.07	0.13	0.65
4	Satakunta	1 399	1 311	1 209	102	0.11	0.19	0.03	0.27	0.61
5	Kanta-Häme	1 037	948	897	51	0.07	0.12	0.02	0.14	0.35
6	Pirkanmaa	1 655	1 492	1 407	85	0.17	0.20	0.04	0.22	0.63
7	Päijät-Häme	938	856	828	28	0.08	0.10	0.02	0.08	0.27
8	Kymenlaakso	699	636	612	23	0.05	0.07	0.02	0.06	0.20
9	South Karelia	516	456	406	50	0.06	0.08	0.01	0.13	0.28
10	South Savo	630	586	546	40	0.12	0.09	0.01	0.09	0.31
11	North Savo	1 535	1 427	1 280	147	0.37	0.25	0.03	0.34	1.00
12	North Karelia	842	792	697	95	0.15	0.14	0.02	0.22	0.54
13	Central Finland	907	808	743	65	0.14	0.13	0.02	0.15	0.44
14	South Ostrobothnia	2 648	2 409	2 030	379	0.39	0.49	0.05	0.96	1.90
15	Ostrobothnia	1 259	1 180	1 090	90	0.20	0.19	0.03	0.22	0.63
16	Central Ostrobothnia	583	549	391	158	0.19	0.16	0.01	0.37	0.73
17	North Ostrobothnia	2 399	2 183	1 574	609	0.45	0.58	0.03	1.47	2.53
18	Kainuu	253	226	174	53	0.06	0.06	0.00	0.12	0.24
19	Lapland	427	401	284	118	0.19	0.13	0.00	0.25	0.58
	**Total**	**22 421**	**20 435**	**18 265**	**2 171**	**3.04**	**3.43**	**0.47**	**5.31**	**12.24**

Table S14 Forest area (km^2). emissions and sequestration by region ($TgCO_2eq\ yr^{-1}$)

**Region code**	**1**	**2**	**4**	**5**	**6**	**7**	**8**	**9**	**10**	**11**	**12**	**13**	**14**	**15**	**16**	**17**	**18**	**19**	**Total**
Region	**Uusi maa**	**Southwest Finland**	**Satakunta**	**Kanta-Häme**	**Pirkanmaa**	**Päijät-Häme**	**Kymen-laakso**	**South Karelia**	**South Savo**	**North Savo**	**North Karelia**	**Central Finland**	**South Ostro-bothnia**	**Ostro-bothnia**	**Central Ostro-bothnia**	**North Ostro-bothnia**	**Kainuu**	**Lapland**	**Total**
Drained peatland area (km^2)	258	326	755	366	1 101	233	302	485	1 095	2 213	3 469	1 726	2 264	754	1 079	8 669	4 726	6 904	36 725
Mineral soil area (km^2)	5 503	6 069	4 539	3 188	8 930	3 969	2 971	3 661	9 638	11 595	11 867	11 697	7 066	4 450	2 363	16 986	11 509	48 404	174 405
Total area (km^2)	5 761	6 394	5 294	3 554	10 031	4 202	3 273	4 147	10 733	13 808	15 335	13 423	9 330	5 204	3 442	25 655	16 235	55 307	211 130
Timber harvest on drained peatland ($TgCO_2eq\ yr^{-}1$)	0.1	0.1	0.3	0.2	0.4	0.1	0.2	0.3	0.6	0.8	1.1	0.6	0.5	0.3	0.2	1.7	0.8	0.5	8.8
Timber harvest on mineral soil ($TgCO_2eq\ yr^{-1}$)	2.2	1.9	1.7	1.6	3.6	1.7	1.4	1.7	4.8	4.4	3.3	4.5	2.0	1.4	0.6	3.2	1.9	3.0	44.7
Timber harvest all soils ($TgCO_2eq\ yr^{-1}$)	2.3	2.0	2.0	1.7	4.0	1.8	1.6	2.0	5.4	5.2	4.5	5.1	2.5	1.6	0.8	4.9	2.7	3.5	53.6
Energywood harvest on drained peatland ($TgCO_2eq\ yr^{-1}$)	0.0	0.0	0.0	0.0	0.1	0.0	0.0	0.0	0.0	0.1	0.1	0.1	0.1	0.1	0.0	0.2	0.1	0.0	1.0
Energywood harvest on mineral soil ($TgCO_2eq\ yr^{-1}$)	0.7	0.5	0.2	0.2	0.6	0.3	0.1	0.2	0.3	0.3	0.2	0.5	0.2	0.3	0.1	0.3	0.1	0.2	5.2
Energywood harvest all soils ($TgCO_2eq\ yr^{-1}$)	0.7	0.6	0.3	0.2	0.6	0.3	0.1	0.2	0.3	0.4	0.3	0.6	0.3	0.4	0.1	0.4	0.1	0.2	6.2
Total harvest. all soils ($TgCO_2eq\ yr^{-1}$)	3.0	2.6	2.3	2.0	4.6	2.1	1.7	2.2	5.7	5.6	4.7	5.7	2.8	2.0	0.9	5.3	2.9	3.8	59.8
CH4 emission ($TgCO_2eq\ yr^{-1}$)	0.0	0.0	0.0	0.0	0.0	0.0	0.0	0.0	0.0	0.0	0.0	0.0	0.0	0.0	0.0	0.1	0.0	0.1	0.4
N2O emission ($TgCO_2eq\ yr^{-1}$)	0.0	0.0	0.0	0.0	0.0	0.0	0.0	0.0	0.0	0.1	0.1	0.1	0.1	0.0	0.0	0.3	0.2	0.3	1.4
Forest emission on drained peatland ($TgCO_2eq\ yr^{-1}$)	0.2	0.2	0.3	0.2	0.5	0.1	0.2	0.3	0.7	1.0	1.4	0.7	0.7	0.4	0.3	2.3	1.1	0.8	11.6
Forest emission on mineral soil ($TgCO_2eq\ yr^{-1}$)	2.8	2.5	2.0	1.8	4.1	2.0	1.5	1.9	5.1	4.7	3.5	5.0	2.2	1.7	0.6	3.5	2.0	3.3	50.0
Forest emission all soils ($TgCO_2eq\ yr^{-1}$)	3.0	2.6	2.3	2.0	4.6	2.1	1.8	2.2	5.8	5.8	4.9	5.7	2.9	2.1	0.9	5.8	3.1	4.1	61.6
Forest sequestration. drained peatland ($TgCO_2eq\ yr^{-1}$)	-0.1	-0.2	-0.3	-0.1	-0.4	-0.1	0.0	0.0	-0.1	-0.4	-0.7	-0.6	-0.9	-0.2	-0.3	-2.0	-1.4	-1.7	-9.4
Forest sequestration. mineral soil ($TgCO_2eq\ yr^{-1}$)	-3.5	-3.9	-2.6	-1.6	-4.9	-2.2	-1.4	-1.7	-4.0	-5.8	-5.9	-6.0	-3.9	-2.7	-1.2	-7.7	-5.7	-15.4	-79.9
Forest sequestration. all soils	-3.6	-4.0	-2.9	-1.7	-5.4	-2.2	-1.4	-1.7	-4.1	-6.2	-6.6	-6.5	-4.8	-2.9	-1.5	-9.7	-7.0	-17.0	-89.3

($TgCO_2eq\ yr^{-1}$)

Forest net emissions drained peatland (TgCO2eq yr-1)	0.07	0.00	0.04	0.14	0.08	0.09	0.22	0.32	0.61	0.65	0.68	0.19	-0.21	0.14	-0.01	0.26	-0.23	-0.82	2.21
Forest net emissions mineral soil (TgCO2eq yr-1)	-0.69	-1.42	-0.61	0.13	-0.79	-0.20	0.15	0.19	1.08	-1.12	-2.38	-0.97	-1.72	-0.98	-0.57	-4.20	-3.72	-12.12	-29.93
Forest net emissions all soils ($TgCO_2eq\ yr^{-1}$)	-0.6	-1.4	-0.6	0.3	-0.7	-0.1	0.4	0.5	1.7	-0.5	-1.7	-0.8	-1.9	-0.8	-0.6	-3.9	-4.0	-12.9	-27.7

Table S15 Waterbody area (km^2) and emissions by region for (TgCO$_2$eq yr^{-1})

Region Code	Region	Total waterbody area (km^2)	N of lakes	Lake area (km^2)	N or river stretches	River area (km^2)	Lake CO_2 emissions (TgCO$_2$eq. yr^{-1})	Lake CH_4 emissions (TgCO$_2$eq. yr^{-1})	Lake emissions (TgCO$_2$eq. yr^{-1})	River CO_2 emissions (TgCO$_2$eq. yr^{-1})	Total waterbody emissions (TgCO$_2$eq. yr^{-1})
1	Uusimaa	488	1 065	454	4 399	34	0.1	0.0	0.1	0.2	0.3
2	Southwest Finland	210	683	175	4 421	35	0.0	0.0	0.0	0.2	0.3
4	Satakunta	458	537	405	3 278	53	0.1	0.0	0.1	0.2	0.3
5	Kanta-Häme	516	817	499	2 238	17	0.1	0.0	0.1	0.1	0.2
6	Pirkanmaa	2 320	2 982	2 284	5 267	36	0.4	0.1	0.4	0.2	0.7
7	Päijät-Häme	1 234	1 083	1 219	2 400	15	0.2	0.0	0.2	0.1	0.3
8	Kymenlaakso	392	743	357	2 110	35	0.1	0.0	0.1	0.1	0.2
9	South Karelia	1 464	1 299	1 448	2 913	15	0.2	0.0	0.2	0.1	0.3
10	South Savo	4 435	4 421	4 414	6 247	21	0.6	0.1	0.7	0.2	0.9
11	North Savo	3 755	3 760	3 704	8 450	51	0.5	0.1	0.6	0.4	1.0
12	North Karelia	3 901	4 965	3 792	10 405	109	0.5	0.1	0.6	0.5	1.1
13	Central Finland	2 937	3 482	2 898	6 951	38	0.4	0.1	0.5	0.3	0.8
14	South Ostrobothnia	574	717	518	9 936	57	0.1	0.0	0.1	0.4	0.5
15	Ostrobothnia	190	529	151	6 239	38	0.0	0.0	0.0	0.3	0.3
16	Central Ostrobothnia	211	474	189	1 772	22	0.0	0.0	0.0	0.1	0.2
17	North Ostrobothnia	2 386	4 461	2 153	15 920	233	0.4	0.1	0.5	1.0	1.4
18	Kainuu	2 494	4 558	2 409	9 271	85	0.4	0.1	0.5	0.5	1.0
19	Lapland	5 933	20 444	5 440	43 772	493	1.0	0.2	1.2	2.3	3.5
	Total	33 896	57 020	32 510	145 989	1 386	5.2	0.9	6.0	7.3	13.4

Table S16 Undrained mires area (km^2) and emissions by region for (TgCO_2eq yr^{-1})

Region Code	Region	Peat production area (km^2)	Productive forested mires area (km^2)	Sedge fens area (km^2)	Other open and sparsely treed fens area (km^2)	Ombro-trophic bogs area (km^2)	Undrained mires total area (km^2)	Wetland total area (km^2)	Undrained mires emission (TgCO_2eq yr^{-1})	Peat production emission (TgCO_2eq)	Total wetland mission (TgCO_2eq yr^{-1})	Undrained mires sink (TgCO_2eq yr^{-1})	Net wetland emission (TgCO_2eq yr^{-1})	Sequestration (TgCO_2eq yr^{-1})
1	Uusimaa	4	75	3	14	52	143	147	0.02	0.01	0.03	-0.02	0.01	0.02
2	Southwest Finland	48	53	11	62	89	215	263	0.05	0.02	0.07	-0.03	0.05	0.03
4	Satakunta	38	68	14	74	137	293	332	0.07	0.14	0.21	-0.04	0.18	0.04
5	Kanta-Häme	6	57	4	25	47	134	140	0.03	0.02	0.05	-0.02	0.03	0.02
6	Pirkanmaa	41	128	8	49	104	289	330	0.06	0.09	0.15	-0.03	0.11	0.03
7	Päijät-Häme	8	47	4	6	15	72	79	0.01	0.01	0.02	-0.01	0.01	0.01
8	Kymenlaakso	16	36	9	19	31	95	111	0.02	0.04	0.06	-0.01	0.05	0.01
9	South Karelia	23	60	6	13	28	107	130	0.02	0.04	0.06	-0.01	0.05	0.01
10	South Savo	34	162	9	33	63	268	301	0.05	0.06	0.11	-0.03	0.07	0.03
11	North Savo	58	257	20	108	102	489	547	0.10	0.13	0.23	-0.06	0.17	0.06
12	North Karelia	45	363	44	288	324	1 020	1 065	0.24	0.03	0.27	-0.12	0.15	0.12
13	Central Finland	77	137	23	81	124	365	442	0.08	0.15	0.23	-0.04	0.19	0.04
14	South Ostrobothnia	151	99	29	171	300	599	750	0.15	0.44	0.59	-0.07	0.52	0.07
15	Ostrobothnia	80	51	16	86	93	245	325	0.07	0.01	0.08	-0.03	0.05	0.03
16	Central Ostrobothnia	56	49	23	238	192	503	559	0.16	0.07	0.23	-0.06	0.16	0.06
17	North Ostrobothnia	233	725	903	2 322	951	4 900	5 133	1.89	0.50	2.39	-0.62	1.77	0.62
18	Kainuu	28	444	200	843	512	2 000	2 028	0.64	0.06	0.70	-0.25	0.45	0.25
19	Lapland	79	1 040	5 273	9 929	2 530	18 772	18 851	8.65	0.14	8.79	-2.45	6.34	2.45
	Total	1026	3 851	6 599	14 362	5 696	30 508	31 534	12.32	1.95	14.27	-3.91	10.36	3.91

Table S17 Relative area (km^2/km^2) and relative emission ($TgCO_2eq/TgCO_2eq$) by region for each land cover class.

Class	Artificial surfaces	Artificial surfaces	Arable land	Arable land	Forest	Forest	Forest	Waterbody	Waterbody	Wetland	Wetland	Wetland
Region code	Relative area	Relative emission	Relative area	Relative emission	Relative area	Relative emission	Relative sequestration	Relative area	Relative emission	Relative area	Relative emission	Relative sequestration
1	0.10	0.78	0.20	0.02	0.63	0.18	0.99	0.05	0.02	0.02	0.00	0.00
2	0.05	0.48	0.27	0.09	0.60	0.38	0.99	0.02	0.04	0.02	0.01	0.01
4	0.06	0.41	0.18	0.10	0.68	0.40	0.99	0.06	0.05	0.04	0.04	0.01
5	0.05	0.34	0.20	0.09	0.68	0.50	0.99	0.09	0.06	0.03	0.01	0.01
6	0.05	0.31	0.12	0.07	0.76	0.53	0.99	0.16	0.07	0.02	0.02	0.01
7	0.05	0.32	0.16	0.07	0.74	0.53	1.00	0.18	0.08	0.01	0.00	0.00
8	0.05	0.39	0.15	0.05	0.72	0.48	0.99	0.08	0.06	0.02	0.02	0.01
9	0.06	0.37	0.10	0.06	0.78	0.48	0.99	0.23	0.07	0.02	0.01	0.01
10	0.03	0.13	0.05	0.04	0.85	0.71	0.99	0.27	0.11	0.02	0.01	0.01
11	0.03	0.16	0.09	0.10	0.80	0.60	0.99	0.19	0.1	0.03	0.02	0.01
12	0.02	0.13	0.04	0.07	0.82	0.62	0.98	0.18	0.14	0.06	0.03	0.02
13	0.03	0.17	0.06	0.05	0.84	0.66	0.99	0.16	0.09	0.03	0.03	0.01
14	0.04	0.19	0.19	0.26	0.68	0.40	0.98	0.04	0.07	0.05	0.08	0.01
15	0.03	0.36	0.17	0.13	0.70	0.43	0.99	0.03	0.06	0.04	0.02	0.01
16	0.04	0.19	0.12	0.29	0.69	0.37	0.96	0.04	0.06	0.11	0.09	0.04
17	0.02	0.35	0.07	0.14	0.70	0.31	0.94	0.07	0.08	0.14	0.13	0.06
18	0.01	0.10	0.01	0.04	0.80	0.56	0.97	0.12	0.17	0.10	0.13	0.03
19	0.01	0.13	0.00	0.03	0.60	0.21	0.87	0.07	0.18	0.20	0.45	0.13
**All regions**	**0.03**	**0.31**	**0.07**	**0.08**	**0.70**	**0.42**	**0.96**	**0.11**	**0.09**	**0.10**	**0.10**	**0.04**

References

Alm J., Shurpali N. J., Minkkinen K., Aro L., Hytönen J., Laurila T., Lohila A., Maljanen M., Martikainen P.J., Mäkiranta P., Penttilä T., Saarnio S., Silvan N., Tuittila E.-S. and Laine J., 2007. Emission factors and their uncertainty for the exchange of CO2, CH4 and N2O in Finnish managed peatlands. Boreal Environment Research 12. 191-209. http://www.borenv.net/BER/archive/pdfs/ber12/ber12-191.pdf

Bastviken, D., Cole, J., Pace, M. and Tranvik, L., 2004. Methane emission from lakes: dependence of lake characteristics, two regional assessments, and a global estimate. Global Biogeochemical Cycles 18(4). GB4009. https://doi.org/10.1029/2004GB002238

Bergström, I., Mäkelä, S., Kankaala, P. and Kortelainen, P. 2007. Methane efflux from littoral vegetation stands of southern boreal lakes: An upscaled regional estimate. Atmospheric Environment 41: 339-351. http://dx.doi.org/10.1016/j.atmosenv.2006.08.014

Haaspuro T. 2013. LUONNIKAS – laskentatyökalu kunnille luontoperäisten kasvihuonekaasujen nielujen ja lähteiden arviointiin. (In Finnish with English abstract.) Novia publikation och produktion. serie/sarja A: Artiklar/Artikkelit 2. Utgivare/julkaisija: Yrkeshögskolan Novia. Fabriksgatan 1. Vasa. Finland. ISBN: 978-952-5839-75-3. https://www.novia.fi/dmsdocument/40

Humborg C., Mörth C.-M., Sundbom M., Borg H., Blenckner T., Giesler R. and Ittekkot V. 2010. CO2 supersaturation along the aquatic conduit in Swedish watersheds as constrained by terrestrial respiration. aquatic respiration and weathering. Global Change Biology 16: 1966–1978. https://doi.org/10.1111/j.1365-2486.2009.02092.x

IPCC, 2006. Eggleston, S., Buendia, L., Miwa, K., Ngara, T. & Tanabe, K. (Ed.). 2006 IPCC Guidelines for National Greenhouse Gas

IPCC 2014. 2013 Supplement to the 2006 IPCC Guidelines for National Greenhouse Gas Inventories: Wetlands. Hiraishi, T., Krug, T., Tanabe, K., Srivastava, N., Baasansuren, J., Fukuda, M. and Troxler, T.G. (eds). Published: IPCC. Switzerland. http://www.ipcc-nggip.iges.or.jp/public/wetlands/index.html.

Juutinen S. Alm J. and Larmola T. 2003. Major implication of the littoral zone for methane release from boreal lakes. Global Biogeochemical Cycles. 17. No. 4. 1117. https://doi.org/10.1029/2003GB002105

Juutinen, S., Rantakari, M., Kortelainen, P., Huttunen, J.T., Larmola, T., Alm, J., Silvola, J. and Martikainen, P.J., 2009. Methane dynamics in different boreal lake types. Biogeosciences 6: 209-233. doi:10.5194/bg-6-209-2009

Karvosenoja, N. 2008. Emission scenario model for regional air pollution. Monographs of the Boreal Environment Research 32. http://hdl.handle.net/10138/39332

Karvosenoja, N., Paunu, V.-V., Savolahti, M., Kupiainen, K., Karppinen, A., Kukkonen, J. and Hänninen, O., 2018. A high-resolution national emission inventory and dispersion modelling – Is population density a sufficient proxy variable? 36th International Technical Meetings (ITM) on Air Pollution Modelling and its Application. Ottawa. Canada. 14.05.2018 - 18.05.2018. https://doi.org/10.1007/978-3-030-22055-6_31

Kortelainen, P., Rantakari, M., Huttunen, J., Mattsson, T., Alm, J., Juutinen, S., Larmola, T., Silvola J, and Martikainen. P., 2006. Sediment respiration and lake trophic state are important predictors of large CO2 evasion from small boreal lakes. Global Change Biology 12: 1554-1567. doi:10.1111/j.1365-2486.2006.01167.x

Lilja, H., Uusitalo, R., Yli-Halla, M., Nevalainen, R., Väänänen, T., and Tamminen, P.2006. Suomen maannostietokanta: Maannoskartta 1:250 000 ja maaperän ominaisuuksia. MTT:n selvityksiä 114. http://urn.fi/URN:ISBN:952-487-019-3

Lilja, H., Uusitalo, R., Yli-Halla, M., Nevalainen, R., Väänänen, T., Tamminen, P. and Tuhtar. J., 2017. Suomen maannostietokanta. Käyttöopas. User’s Guide for Finnish Digital Soil Map (In Finnish) Luonnonvara- ja biotalouden tutkimus 6/2017. http://urn.fi/URN:ISBN:978-952-326-357-4

Lounasheimo J., Karhinen S., Grönroos J., Savolainen H., Forsberg T., Munther J., Petäjä J. and Pesu J. 2020. The calculation of the greenhouse gas emissions of Finnish municipalities. Reports of the Finnish Environment Institute 25/2020. In Finnish only. Aggregated emission data and English description available at: https://hiilineutraalisuomi.fi/en-US/Emissions_and_indicators/Municipalities_and_regions_greenhouse_gas_emissions

Mäkisara, K., Katila, M. and Peräsaari, J. 2022. The Multi-Source national forest inventory of Finland — methods and results 2017 and 2019. Natural resources and bioeconomy studies
90/2022. Natural Resources Institute Finland. Helsinki. http://urn.fi/URN:ISBN:978-952-380-538-5

Minkkinen, K. and Ojanen, P., 2013. Pohjois-Pohjanmaan turvemaiden kasvihuonekaasutaseet. (In Finnish. Metlan työraportteja; Vol. 258). Metsäntutkimuslaitos. http://www.metla.fi/julkaisut/workingpapers/2013/mwp258.htm

Minkkinen, K., Ojanen, P., Penttilä, T., Aurela, M., Laurila, T., Tuovinen, J.-P. and Lohila, A., 2018. Persistent carbon sink at a boreal drained bog forest. Biogeosciences 15: 3603-3624. https://doi.org/10.5194/bg-15-3603-2018

Minkkinen, K., Ojanen, P., Koskinen, M. and Penttilä, T., 2020. Nitrous oxide emissions of undrained, forestry-drained, and rewetted boreal peatlands. Forest Ecology and Management Vol 478. https://doi.org/10.1016/j.foreco.2020.118494

Minunno, F., Peltoniemi, M., Launiainen, S., Aurela, M., Lindroth, A., Lohila, A., Mammarella, I., Minkkinen, K. and Mäkelä, A., 2016. Calibration and validation of a semi-empirical flux ecosystem model for coniferous forests in the Boreal region. Ecological Modelling 341:37-52. https://doi.org/10.1016/j.ecolmodel.2016.09.020

Minunno, F., Peltoniemi, M., Härkönen, S., Kalliokoski, T., Makinen, H. and Mäkelä, A., 2019. Bayesian calibration of a carbon balance model PREBAS using data from permanent growth experiments and national forest inventory. Forest Ecology and Management 440:208-257. https://doi.org/10.1016/j.foreco.2019.02.041

National Land Survey of Finland. 2022. Regions and their areas 2022. Available at https://www.maanmittauslaitos.fi/sites/maanmittauslaitos.fi/files/attachments/2022/01/Vuoden_2022_pinta-alatilasto_kunnat_maakunnat.pdf . Accessed 1.10.2022.

Nykänen, H., Silvola, J., Alm, J. and Martikainen, P. 1996. Fluxes of greenhouse gases CH4, CO2 and N2O on some peat mining areas in Finland. In: Laiho, R., Laine, J. and Vasander, H. (eds.) (1996). Northern Peatland in global climate change. (Proceedings of the International Workshop held in Hyytiälä. Finland. 8-12 October 1995. The Finnish Research Programme on Climate Change – SILMU). Publications of the Academy of Finland 1/96. 141-147.

Ojanen, P., Minkkinen, K., Alm, J., Penttilä, T., 2010. Soil–atmosphere CO2. CH4 and N2O fluxes in boreal forestry-drained peatlands. Forest Ecology and Management 260(3): 411-421. https://doi.org/10.1016/j.foreco.2010.04.036

Ojanen. P., Minkkinen. K., Penttilä. T., 2013. The current greenhouse gas impact of forestry-drained boreal peatlands. Forest Ecology and Management 289:201-208. https://doi.org/10.1016/j.foreco.2012.10.008

Ojanen, P., Penttilä, T., Tolvanen, A., Hotanen J.-P., Saarimaa, M., Nousiainen, H., Minkkinen, K., 2019. Long-term effect of fertilization on the greenhouse gas exchange of low-productive peatland forests. Forest Ecology and Management 432:786- 798. https://doi.org/10.1016/j.foreco.2018.10.015.

Paunu V.-V., Karvosenoja N., Savolahti M. and Kupiainen K. 2013. High quality spatial model for residential wood combustion emissions. 16th IUAPPA World Clean Air Congress. Cape Town. South Africa. 29 September - 4 October 2013. 4 pp. 11.

Statistics Finland, 2022. Greenhouse gas emissions in Finland 1990 to 2020. National Inventory Report under the UNFCCC and the Kyoto Protocol March 15, 2022. Available: https://www.stat.fi/static/media/uploads/tup/khkinv/fi_nir_eu_2020_2022-03-15.pdf

Statistics Finland. 2022a. Population data by region 31.12.2020. Available at https://www.tilastokeskus.fi/tup/suoluk/suoluk_vaesto_en.html Accessed 1.10.2022

Statistics Finland. 2023. NUTS regional classification. Available at https://www.stat.fi/en/luokitukset/nuts/ Accessed 14.6.2023.

Sved, J. and Koistinen, A. (eds.). 2015. Metsänhoidon suositukset kannattavaan metsätalouteen. työopas. In Finnish (Best Practices for Profitable Forest Management). Publications by Tapio Group HTTP://TAPIO.FI Available in Finnish https://tapio.fi/wp-content/uploads/2015/09/MHS_Kannattava_metsatalous_201500814.pdf Accessed 30.6.2020.

Tomppo, E., Katila, M., Mäkisara, K., and Peräsaari, J., 2014. The Multi-source National Forest Inventory of Finland – methods and results 2011. 224 pp. http://urn.fi/URN:ISBN:978-951-40-2516-7

Tuomi, M., Thum, T., Järvinen, H., Fronzek, S., Berg, B., Harmon, M., Trofymow, J.A., Sevanto, S. and Liski. J., 2009. Leaf litter decomposition – Estimates of global variability based on Yasso07 model. Ecol. Modell. 220:3362–3371. https://doi.org/10.1016/j.ecolmodel.2009.05.016

Turunen, J., Tomppo, E., Tolonen, K., Reinikainen, A., 2002. Estimating carbon accumulation rates of undrained mires in Finland – application to boreal and subarctic regions. The Holocene 12 (1): 69-80. https://journals.sagepub.com/doi/abs/10.1191/0959683602hl522rp
